# Optical changes and axial elongation in children wearing orthokeratology lenses of smaller back optic zone diameter: a systematic review and meta-analysis

**DOI:** 10.7717/peerj.20928

**Published:** 2026-03-11

**Authors:** Haobo Fan, Xiaoxiao Wu, Jia Yu, Qiumei Wei, Xuemin Zhang, Airui Xie, Junguo Duan

**Affiliations:** 1 Eye College, Chengdu University of Traditional Chinese Medicine, Chengdu, Sichuan, China; 2 Department of Optometry and Pediatric Ophthalmology, Ineye Hospital of Chengdu University of Traditional Chinese Medicine, Chengdu, Sichuan, China; 3 Key Laboratory of Sichuan Province Ophthalmopathy Prevention & Cure and Visual Function Protection with TCM Laboratory, Chengdu, Sichuan, China; 4 Retinal Image Technology and Chronic Vascular Disease Prevention & Control and Collaborative Innovation Center, Chengdu, Sichuan, China

**Keywords:** Meta-analysis, Myopia, Orthokeratology, Higher-order aberrations

## Abstract

**Objectives:**

To evaluate the effect of orthokeratology lenses (Ortho-K) with smaller back optic zone diameters (BOZD) on axial elongation and ocular higher-order aberrations in children and adolescents.

**Methods:**

PubMed, the Cochrane Library, Embase, CNKI, and Web of Science databases were comprehensively searched for relevant studies. The Cochrane risk-of-bias tool and the Newcastle-Ottawa Scale were used to assess the risk of bias in the included studies. RevMan 5.4 was applied for meta-analysis, and STATA 17.0 was employed for meta-regression, sensitivity analysis, and publication bias assessment. This study was registered at PROSPERO (registration number CRD42024538742).

**Results:**

Seventeen studies were included, comprising nine RCTs and eight cohort studies, with 1,548 participants in the smaller BOZD group and 1,570 in the conventional BOZD group. Meta-analysis shows that the smaller BOZD group has a lower axial elongation than the conventional BOZD group at 6 months, 12 months, and 24 months following Ortho-K treatment, with reductions of −0.07 mm (95% CI [−0.09, −0.04] *P* < 0.00001), −0.12 mm (95% CI [−0.13, −0.10] *P* < 0.00001), and −0.14 mm (95% CI [−0.19, −0.08]; *P* < 0.00001), respectively. Furthermore, compared to the conventional BOZD group, the smaller BOZD group result in a smaller treatment zone diameter (TZD) (MD: −0.49; 95% CI [−0.62, −0.36] *P* < 0.00001) and increased root mean square (RMS) higher-order aberrations (MD: 0.20; 95% CI [0.16–0.24]; *P* < 0.00001), RMS spherical aberration (MD: 0.17; 95% CI [0.12–0.21]; *P* < 0.00001) and RMS coma (MD = 0.10, 95% CI [0.02–0.18]; *P* < 0.0001) in children.

**Conclusions:**

Our meta-analysis revealed that Ortho-K lenses with smaller BOZD exhibited less axial elongation in children compared with conventional Ortho-K lenses. This effect may be related to the reduction of TZD and the increase of higher-order aberrations (HOAs) caused by the smaller BOZD design.

## Introduction

Over the past few decades, the incidence and severity of myopia have consistently increased (Vitale, Sperduto & Ferris, 2009; Jonas et al., 2021), evolving into an increasingly prominent global public health issue (Dolgin, 2015). It is projected that by 2050, the number of individuals with myopia will rise to nearly 5 billion, with 20% suffering from high myopia (Holden et al., 2016). However, due to variations in regional, environmental, and societal factors, the global distribution of myopia incidence is uneven, with higher prevalence rates observed in East Asia, particularly in China (Pan, Ramamurthy & Saw, 2012). In China, there are currently an estimated 300 million people with myopia, and the overall myopia rate of children and adolescents is 53.6%, among which primary and secondary school students, middle school students, and senior high school students are 36.0%, 71.6%, and 81.0% respectively (Zhu et al., 2023). As myopia progresses, it results in axial elongation (AE) and eyeball dilation, thereby increasing the risk of complications such as cataracts, myopic macular degeneration, staphyloma, retinal detachment, and glaucoma (Holden et al., 2016; Smith, 2013). Considering that myopia typically manifests between the ages of 7 and 10, early intervention during childhood and adolescence is crucial for effective myopia management (Smith, 2013).

Orthokeratology (Ortho-K) lenses serve as one of the classical optical interventions with proven efficacy in controlling myopia in children (Huang et al., 2016; Guan et al., 2020). Although the precise mechanisms underlying their effect on myopia control remain incompletely understood (Vincent et al., 2021), current studies suggest that they may be associated with the induced peripheral retinal myopic defocus shift (Huang et al., 2022a; Huang et al., 2022b; Kang et al., 2021; Li et al., 2023a; Li et al., 2023b; Li et al., 2023c; Li et al., 2023d) and alterations in ocular optical quality (Philip et al., 2014). Recent investigations have shown that, compared to conventional corneal reshaping lens designs, those incorporating a smaller back optic zone diameter (BOZD) can more effectively retard AE in myopic children (Guo et al., 2023a; Guo et al., 2023b; Paune et al., 2021; Li et al., 2023a; Li et al., 2023b; Li et al., 2023c; Li et al., 2023d). Despite existing studies providing evidence-based support for the role of Ortho-K lenses with a smaller BOZD in inhibiting axial elongation in myopic children (Gu et al., 2024; Zhou et al., 2024; Yang et al., 2025).

However, these studies (Zhou et al., 2024; Yang et al., 2025) have not yet been able to assess the association between the higher-order aberrations (HOAs) changes attributable to this new design and the efficacy of myopia control, nor have they further clarified the previously reported controversies concerning alterations in spherical aberration (SA) and coma (Li et al., 2023a; Li et al., 2023b; Li et al., 2023c; Li et al., 2023d; Guo et al., 2023a; Guo et al., 2023b). On the other hand, for this novel design of Ortho-K lenses, such studies (Gu et al., 2024; Zhou et al., 2024) lack long-term efficacy evaluations with follow-up periods exceeding 12 months. In addition, these studies (Zhou et al., 2024; Yang et al., 2025) have also failed to investigate the sources of heterogeneity among the outcome measures included in the meta-analyses. These gaps impose certain limitations on the current body of relevant research.

In view of these deficiencies in the existing literature, the present study aims, through meta-analysis, to compare the effects of smaller BOZD designs on AE and HOAs in children’s eyes, thereby providing insights for the selection and application of Ortho-K lenses with a reduced BOZD in myopia control.

## Materials & Methods

This systematic review was conducted in accordance with the Preferred Reporting Items for Systematic Reviews and Meta-Analyses (PRISMA) (Moher et al., 2015) guidelines and has been registered in the International Prospective Register of Systematic Reviews (PROSPERO) under the number CRD42024538742. The PRISMA 2020 checklist is provided in File S1.

### Search strategy

Two researchers (Haobo Fan and Xiaoxiao Wu) conducted a comprehensive search of PubMed, the Cochrane Library, Embase, CNKI, and Web of Science databases to collect relevant studies on the effects of smaller BOZD Ortho-K lenses on myopia control in children. The search spanned the period from the inception of the databases to November 15, 2025, and included studies published in both English and Chinese. The English search terms used were orthokeratology, axial elongation, and back optic zone diameters. These terms were adapted for use in various databases and websites. In addition to the identified studies and relevant systematic reviews, additional studies were included by screening the reference lists of relevant studies and systematic reviews.

### Study selection

This study was guided by the PICOS framework: (P) Population: individuals with myopia ranging from 0 to −6.00 D and astigmatism ranging from 0 to −1.50 D, aged between 7 and 16 years. (I) Intervention/Exposure: Smaller BOZD Ortho-K lenses (BOZD ≤ 5.5 mm). (C) Comparison: Conventional Ortho-K lenses (BOZD ≥ 6.0 mm). (O) Outcomes: AE, treatment zone diameter (TZD), root mean square (RMS) HOAs, RMS SA, and RMS coma during the intervention period. (S) Study design: randomized controlled trials (RCTs) or cohort studies published as full-length articles in English or Chinese. Among RCTs and cohort studies, we excluded studies that (1) did not meet the inclusion criteria; (2) were unavailable or from which complete data could not be extracted; (3) were republished literature; (4) were literature in languages other than English or Chinese.

### Filtrate the articles

Two researchers (Haobo Fan and Xiaoxiao Wu) independently conducted the literature search using a predefined retrieval strategy and compiled their respective search results. The initial screening phase involved excluding duplicate records, case reports, review articles, and other publications deemed irrelevant based on title review, ensuring consistency with the study’s objectives. A thorough evaluation of full texts was conducted, adhering strictly to predefined inclusion and exclusion criteria to assess eligibility for inclusion. The two researchers (Haobo Fan and Xiaoxiao Wu) mutually verified the final selection of the study. If significant heterogeneity was observed among the included studies, a third researcher (Junguo Duan) was consulted to resolve discrepancies and determine final inclusion.

### Data extraction

Essential study characteristics were extracted from all eligible publications, including the authors’ names, study location (country or area), publication year, study design, participant age range, sample size, intervention protocols, study duration, and measured outcomes, to ensure comprehensive data collection for subsequent analysis. This study systematically extracted the baseline-to-endpoint differences in AE, TZD, RMS HOAs, RMS SA, and RMS coma to evaluate the effects of smaller BOZD Ortho-K lenses on children’s ocular optical changes and axial elongation. All extracted data were documented in a standardized Excel spreadsheet (Microsoft Corporation, Redmond, WA, USA) to ensure systematic data management and facilitate subsequent statistical analysis. When available, pre-calculated change values were directly obtained from original studies. All continuous variables were reported as mean ± standard deviation (*SD*); when presented as interquartile ranges or 95% confidence intervals, the appropriate conversions were performed (Wan et al., 2014). For longitudinal studies, data were extracted at predefined time points (6, 12, and 24 months) to allow time-specific comparisons and ensure temporal consistency across studies. Data extraction was independently performed by two researchers (Haobo Fan and Xiaoxiao Wu), and any discrepancies were resolved through discussion until consensus was reached or by consulting a third researcher (Junguo Duan).

### Risk of bias assessment

A comprehensive evaluation of methodological quality was conducted using validated assessment tools appropriate for each study design. For RCTs, the Cochrane Risk of Bias tool (RoB 2.0) was employed to systematically assess randomization processes, allocation concealment, blinding procedures, outcome reporting completeness, and other potential sources of bias. Cohort studies were evaluated using the Newcastle-Ottawa Scale (NOS), with particular attention to participant selection criteria, group comparability, and ascertainment of outcomes and exposures. Based on established quality thresholds, cohort studies achieving an NOS score of 7 or higher were considered methodologically sound. Two researchers (Haobo Fan and Xiaoxiao Wu) independently evaluated the quality of the included studies, and any disagreements were resolved through discussion with a third researcher (Junguo Duan) to reach a consensus.

### Statistical analysis

The meta-analysis was conducted using Review Manager 5.4 software (Cochrane Collaboration). Mean, SD, mean differences (MD), and 95% confidence intervals (CI) were calculated and used as effect measures for changes in AE, TZD, RMS HOAs, RMS SA, and RMS coma. Heterogeneity was assessed using the chi-square test based on *Q* and *I*^2^ statistics. If no significant heterogeneity was observed (*P* > 0.10, *I*^2^ < 50%), a fixed-effects model was applied; otherwise, a random-effects model was used.

Meta-regression, sensitivity analysis, and publication bias assessment were performed using STATA 17.0 (StataCorp, College Station, TX, USA). Sensitivity analysis was conducted using the leave-one-out method to investigate heterogeneity further and evaluate the robustness of the pooled estimates. The results were considered robust if the overall effect size remained statistically unchanged upon sequential exclusion of individual studies. Analysis of potential sources of heterogeneity in the meta-analysis was performed using the leave-one-out method and meta-regression. After confirming, using the leave-one-out method, that the source of between-study heterogeneity was not attributable to any single included study, meta-regression was employed to identify potential factors contributing to heterogeneity.

Funnel plots and Egger’s test were used to assess publication bias. When the funnel plot was asymmetric, or the two-tailed *P-*value from Egger’s test was < 0.05, the outcome measure was considered to have a potential for the presence of publication bias. For outcome measures with evidence of publication bias, the non-parametric trim-and-fill method was applied to evaluate the impact of publication bias on the meta-analytic results.

## Results

### Search strategy

**Figure 1:**
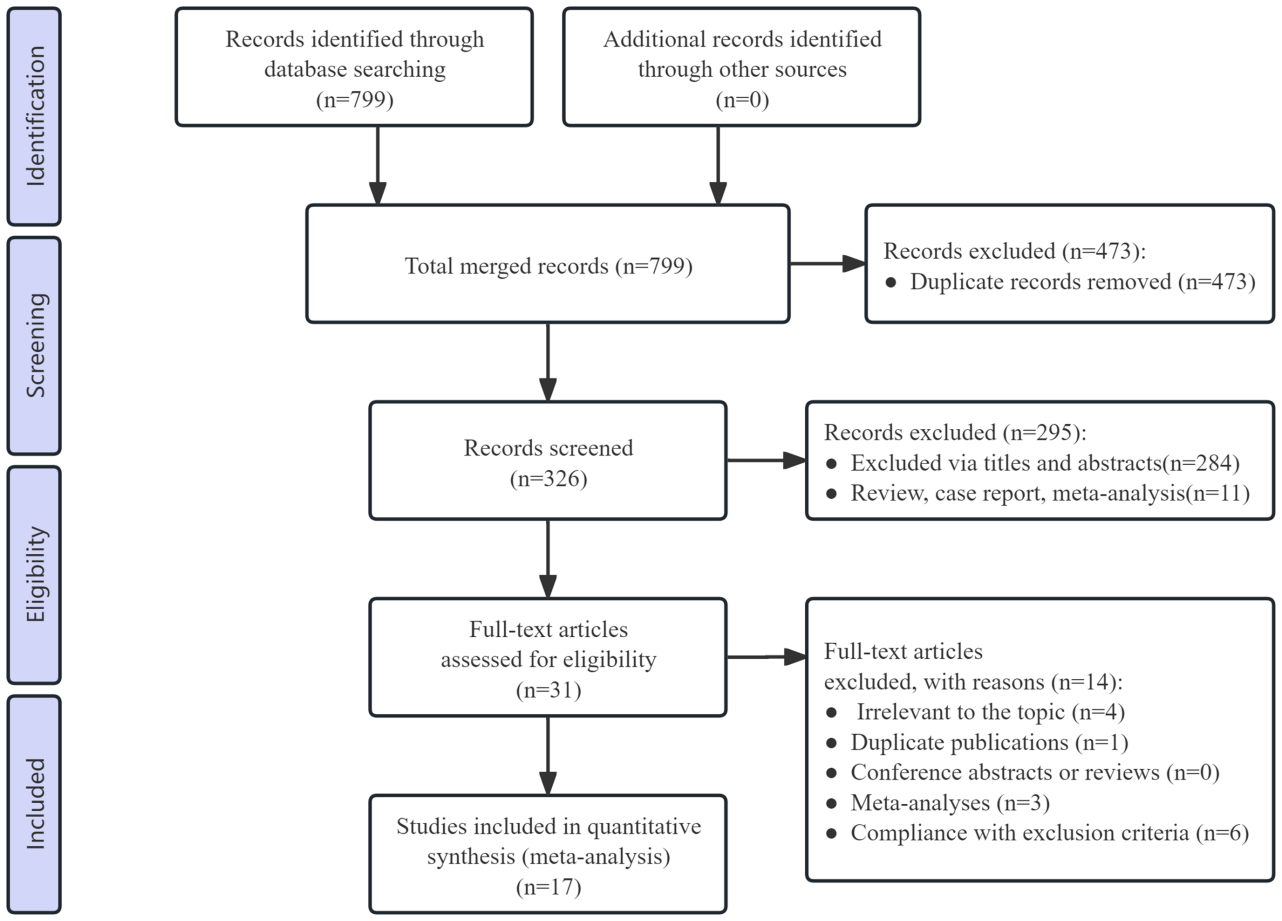
Preferred reporting items for systematic reviews and meta-analyses (PRISMA) flow diagram of the study process.

A total of 799 studies were identified in the initial screening process. After removing duplicates, 326 records remained for screening. After screening the titles and abstracts, 295 irrelevant records were excluded. After assessing the full texts of the remaining 31 records, 14 studies were excluded because they did not meet the inclusion criteria. Finally, 17 studies (nine RCTs and eight cohort studies) were included in the meta-analysis (Wu et al., 2024; Li et al., 2023a; Li et al., 2023b; Li et al., 2023c; Li et al., 2023d; Xu et al., 2023; Tang et al., 2024; Guo et al., 2023a; Guo et al., 2023b; Zhang et al., 2023; Ding et al., 2024; Paune et al., 2021; Tang et al., 2022; Wang et al., 2025; Tan et al., 2025; Li et al., 2025a; Li et al., 2025b; Li et al., 2025c; Jing, Yan & Wan, 2025; Sun et al., 2025). The study selection process is illustrated in a PRISMA flow diagram (Fig. 1).

### Study characteristics and risk of bias assessment

**Figure 2:**
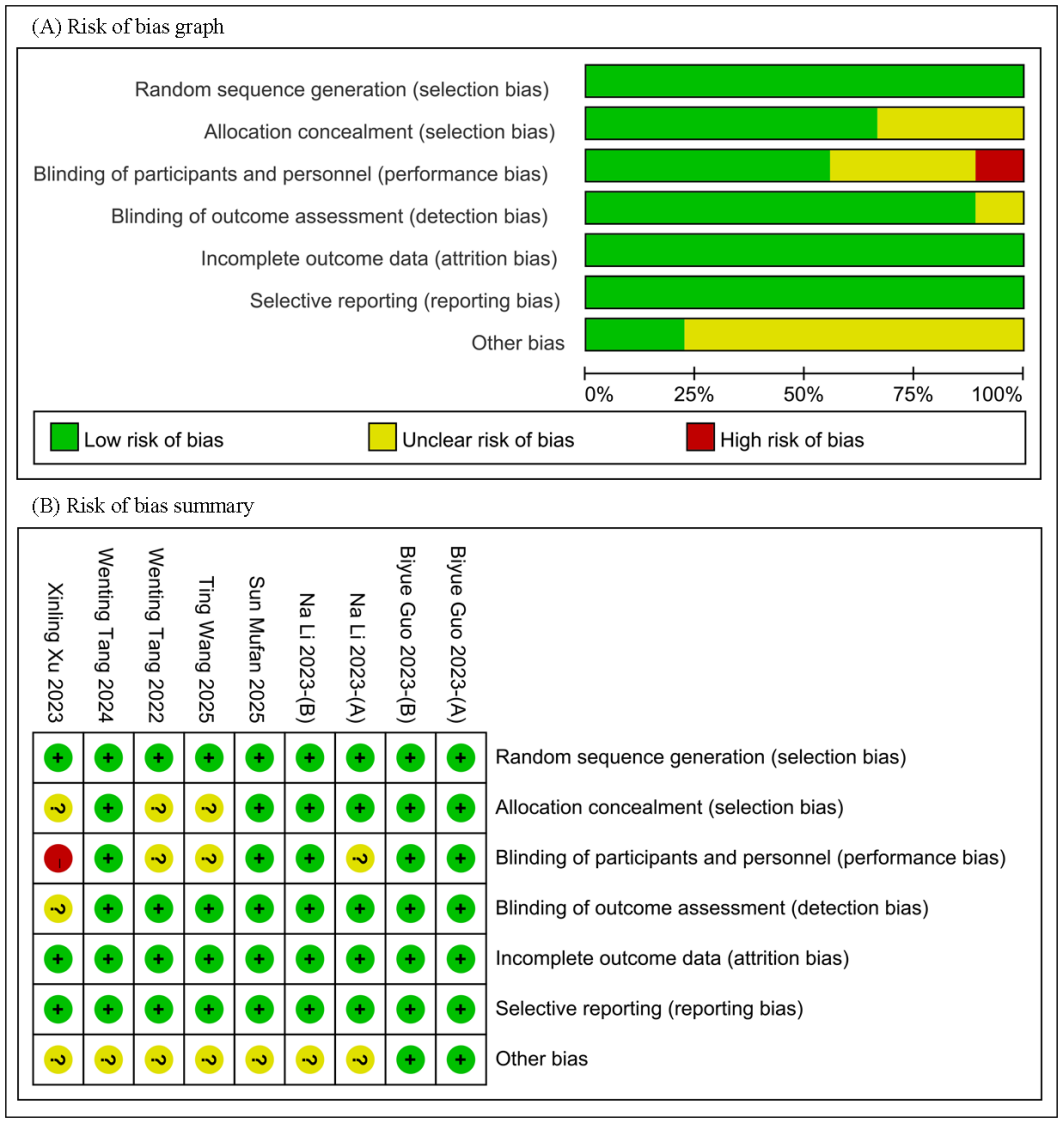
Risk-of-bias assessments of the included studies. (A) Risk of bias graph. (B) Risk of bias summary.

A total of 3,118 myopic children aged 7 to 16 years were enrolled, including 1,548 in the smaller BOZD group and 1,570 in the conventional BOZD group. The studies were conducted in the following countries or regions: China (*n* = 11), Hong Kong (*n* = 2), and Spain (*n* = 1). Among the RCTs, only two had a low risk of bias, while the others had varying degrees of risk of bias. The cohort studies were generally of high quality, with scores of at least 7 out of 9 (Fig. 2, Tables 1–2).

**Table 1:** Characteristics of the studies included in the meta-analysis.

Study (Author, Year)	Country or area	Study design	Ortho-k lenses design	BOZD	Age (years)	Sample size	Baseline		Follow-up duration	Outcomes
							Diopter (D)	Axial length (mm)		
Guo et al. (2023a)	Hong Kong	RCT	KATT BE Free	5 mm	9.03 ± 1.25	22	−2.69 ± 0.89	24.65 ± 0.79	6 m, 12 m, 24 m	AE, TZD
				6 mm	9.31 ± 0.91	23	−2.27 ± 0.85	24.24 ± 0.69		
Guo et al. (2023b)	Hong Kong	RCT	KATT BE Free	5 mm	9.03 ± 1.25	22	−2.69 ± 0.89	24.65 ± 0.79	6 m, 24 m	RMS HOAs, RMS SA, RMS coma
				6 mm	9.32 ± 0.93	23	−2.31 ± 0.84	24.24 ± 0.69		
Li et al. (2025a)	China	Cohort study	CRT	5.0 mm	9.42 ± 1.04	66	2.43 ± 0.79	/	12 m	TZD
				6.0 mm	9.58 ± 1.04	67	2.63 ± 0.76	/		
Paune et al. (2021)	Spain	Cohort study	DRL	≤ 5 mm	13.41 ± 1.25	35	−2.80 ± 1.37	24.61 ± 0.83	12 m	AE
				>5 mm	13.27 ± 1.50	36	−3.41 ± 1.51	24.69 ± 0.94		
Wu et al. (2024)	China	Cohort study	*α*Ortho-K	5.0 mm	9.59 ± 1.45	29	−2.36 ± 0.90	24.33 ± 0.69	6 m, 12 m	AE
				5.4 mm	9.83 ± 1.32	30	−2.48 ± 1.06	24.36 ± 0.51		
				6.0 mm	10.08 ± 1.61	77	−2.35 ± 0.96	24.42 ± 0.68		
Jing, Yan & Wan (2025)	China	Cohort study	CRT/ Euclid	5.0/5.2mm	9.82 ± 2.06	806	−3.31 ± 1.30	24.54 ± 0.83	6 m, 12 m, 24 m	AE, TZD
				6.0/6.2 mm	9.75 ± 1.74	806	−3.31 ± 1.37	24.46 ± 0.79		
Li et al. (2023a)	China	RCT	DRL	5.0 mm	9.00 [8, 11]	24	−2.38 [−4.00, −1.25]	24.67 ± 0.60	12 m	AE, TZD
			Euclid	6.2 mm	9.50 [8, 11]	26	−2.25 [−4.00, −1.00]	24.63 ± 0.78		
Li et al. (2023b)	China	RCT	DRL	5.0 mm	10.65 ± 1.80	46	−2.63 ± 0.85	–	6 m, 12 m	AE, TZD, RMS HOAs, RMS SA, RMS coma
			Euclid	6.2 mm	10.40 ± 1.64	44	−2.67 ± 0.91	–		
Tan et al. (2025)	China	Cohort study	CRT	5.0 mm	9.1 ± 1.2	22	−2.69 ± 0.89	24.65 ± 0.79	24 m	RMS SA
				6.0 mm	9.2 ± 1.1	58	−2.61 ± 0.98	24.40 ± 0.84		
Sun et al. (2025)	China	RCT	DRL	5.0 mm	10.45 ± 1.99	33	−2.56 ± 0.87	24.65 ± 0.75	12 m	AE, TZD
			Euclid	6.2 mm	11.00 ± 2.26	33	−2.89 ± 1.17	24.85 ± 0.87		
Wang et al. (2025)	China	RCT	CRT	5.0 mm	10.06 ± 1.14	33	−2.83 ± 0.78	24.46 ± 0.91	12 m	AE
				6.0 mm	10.06 ± 1.14	33	−2.79 ± 0.85	24.45 ± 0.91		
Tang et al. (2022)	China	RCT	KATT BE Free	5.0 mm	11.76 ± 1.98	41	−3.41 ± 1.12	24.82 ± 0.34	6 m, 12 m	AE, RMS HOAs
				6.0 mm	11.68 ± 2.22	44	−3.37 ± 1.20	24.77 ± 0.38		
Tang et al. (2024)	China	RCT	KATT BE Free	5.0 mm	11.35 ± 2.08	20	−3.23 ± 0.78	24.85 ± 0.65	6 m, 12 m	AE, RMS HOAs
				5.5 mm	10.48 ± 2.18	21	−2.89 ± 0.63	24.67 ± 0.71		
				6.0 mm	11.62 ± 1.91	21	−3.33 ± 0.76	24.91 ± 0.73		
Ding et al. (2024)	China	Cohort study	CRT	5.0 mm	9.02 ± 1.67	86	−2.24 ± 0.98	24.39 ± 0.78	12 m	AE
				6.0 mm	9.15 ± 1.51	60	−2.00 ± 0.99	24.35 ± 0.79		
Xu et al. (2023)	China	RCT	CRT	5.0 mm	10.40 ± 1.01	30	−2.88 ± 0.42	24.62 ± 0.50	6 m, 12 m	AE, TZD
				6.0 mm	10.40 ± 1.03	30	−2.89 ± 0.43	24.64 ± 0.49		
Li et al. (2023d)	China	Cohort study	CRT	5.0 mm	9.67 ± 1.93	147	−2.86 ± 1.62	24.68 ± 0.89	6 m, 12 m	AE, TZD, RMS HOAs, RMS SA, RMS coma
				6.0 mm	9.33 ± 1.77	154	−2.60 ± 1.24	24.65 ± 0.76		
Zhang et al. (2023)	China	Cohort study	DRL	5.0 mm	10.63 ± 1.76	28	−2.84 ± 0.98	24.86 ± 0.71	12 m	AE
				6.2 mm	9.93 ± 1.63	42	−2.68 ± 0.90	24.92 ± 0.77		

**Notes.**

RCT randomized controlled trial DRL double reservoir lens BOZD back optic zone diameters AE Axial elongation TZD treatment zone diameter RMS root-mean-square HOAs higher-order aberrations Coma comatic aberration SA spherical aberration

**Table 2:** Quality assessment of cohort studies included in the meta-analysis using Newcastle-Ottawa Quality Assessment Scale.

Study	Selection				Comparability of cohorts	Exposure			NOS score
	Exposed cohort representative	Non-exposed cohort selection	Exposure ascertainment	Outcome not present at start		Assessment	Follow-up length	Follow-up adequacy	
Li et al. (2025a)	1	1	1	1	1	1	1	1	8
Paune et al. (2021)		1	1	1	1	1	1	1	7
Wu et al. (2024)	1	1	1	1	2	1	1	1	9
Jing, Yan & Wan (2025)	1	1	1	1	2	1	1	1	9
Tan et al. (2025)	1	1	1	1	1	1	1	1	8
Ding et al. (2024)	1	1	1	1	2	1	1	1	9
Li et al. (2023d)	1	1	1	1	2	1	1	1	9
Zhang et al. (2023)	1	1	1	1	1	1	1	1	8

**Notes.**

NOS Newcastle-Ottawa Scale

### Meta-analysis results

#### Axial elongation

**Figure 3:**
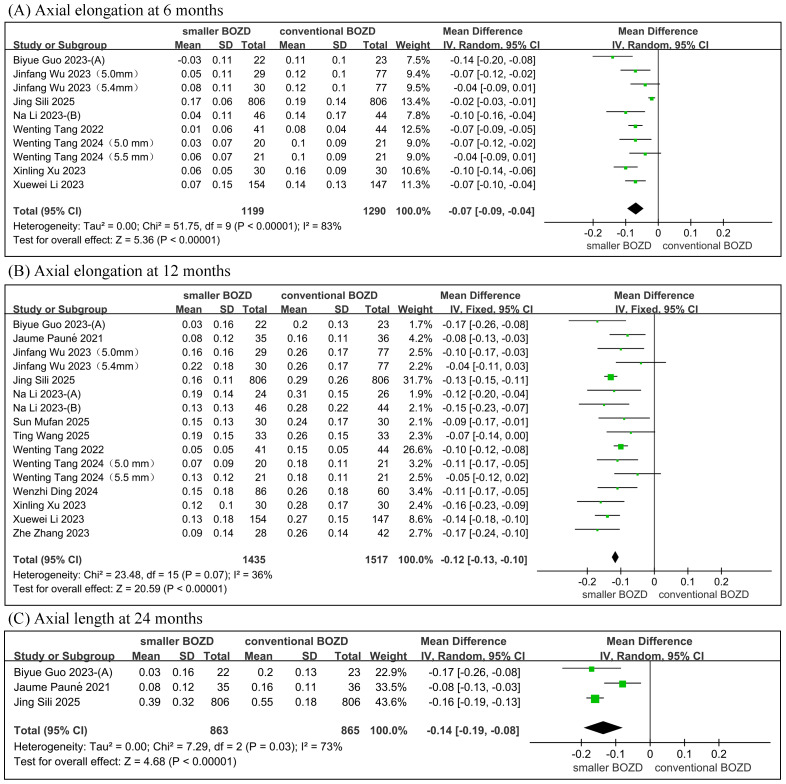
Meta-analysis of the AE between the smaller BOZD Ortho-K lenses group and the conventional Ortho-K lenses group. (A) The forest plot of the AE at 6 months. (B) The forest plot of the AE at 12 months.

The meta-analysis revealed that, compared to the conventional Ortho-K lenses group, the smaller BOZD Ortho-K lenses group showed significantly less AE with between-group differences of −0.07 mm (95% CI [−0.09, −0.04]; *P* < 0.00001) at 6 months, −0.12 mm (95% CI [−0.13, −0.10]; *P* < 0.00001) at 12 months, and −0.14 mm (95% CI [−0.19, −0.08]; *P* < 0.00001) at 24 months, while significant heterogeneity was observed at 6-month (*I*^2^ = 83%) and 24-month (*I*^2^ = 73%) follow-ups but not at 12-month follow-up (*I*^2^ = 36%) (Fig. 3).

### Treatment zone diameter

**Figure 4:**
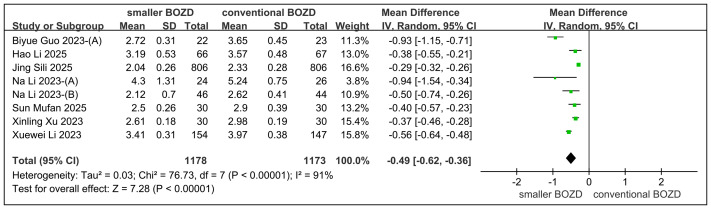
Meta-analysis of the TZD between the smaller BOZD Ortho-K lenses group and the conventional Ortho-K lenses group.

The meta-analysis demonstrated that the smaller BOZD group exhibited significantly less change in TZD compared to the conventional BOZD group, with between-group differences of −0.49 mm (95% CI [−0.62, −0.36]; *P* < 0.00001), while significant heterogeneity was observed (*I*^2^ = 91%) (Fig. 4).

### Higher-order aberrations, spherical aberration, and coma

**Figure 5:**
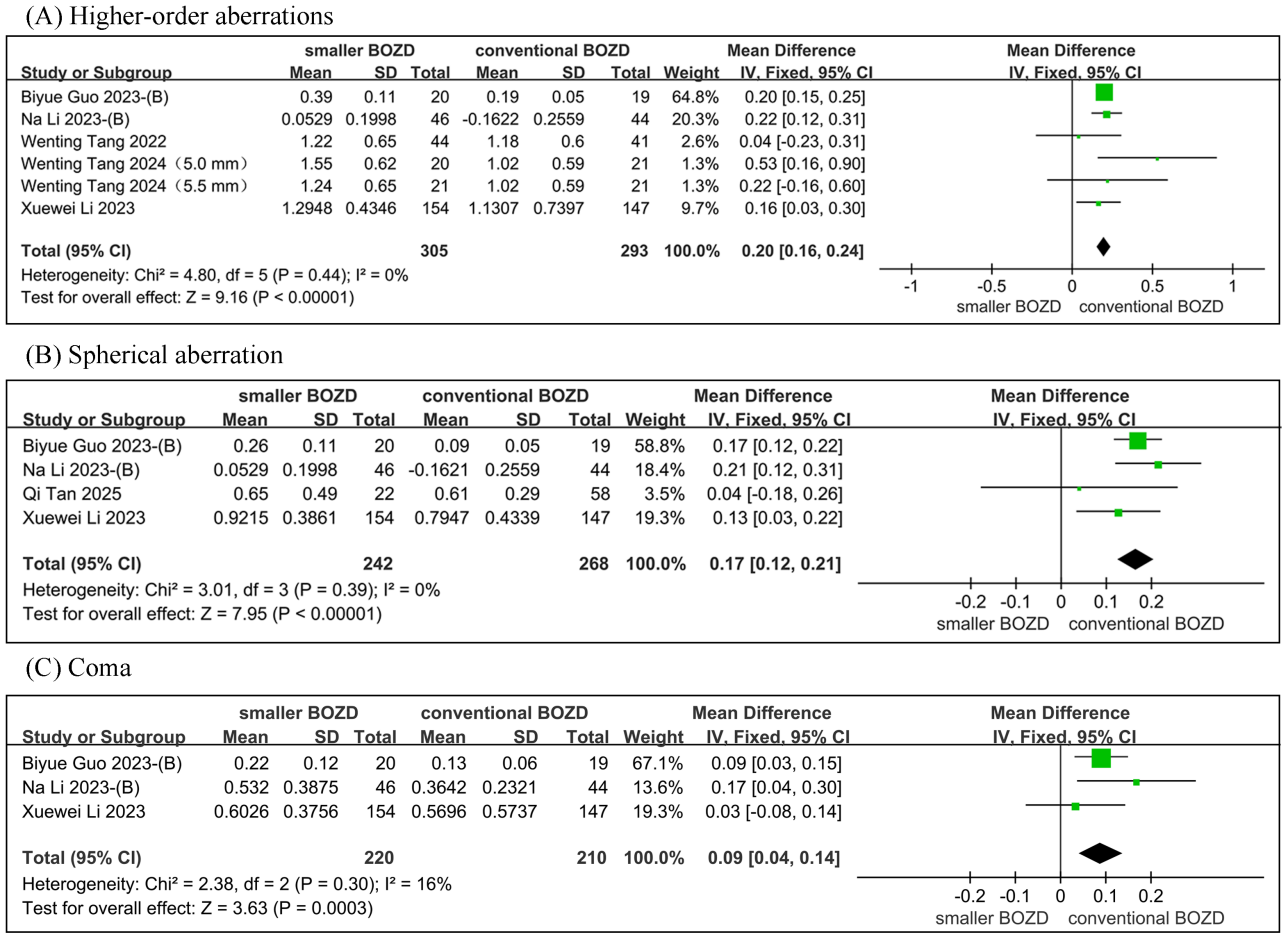
Meta-analysis of the HOAs between the smaller BOZD Ortho-K lenses group and the conventional Ortho-K lenses group. (A) The forest plot of the RMS HOAs. (B) The forest plot of the RMS SA. (C) The forest plot of the RMS coma.

The meta-analysis demonstrated significantly higher RMS HOAs (MD = 0.20, 95% CI [0.16–0.24]; *P* < 0.00001), RMS SA (*MD* = 0.17, 95% CI [0.12–0.21]; *P* < 0.00001), and RMS coma (MD = 0.09, 95% CI [0.04–0.14]; *P* = 0.0003) in the small BOZD group compared to the conventional BOZD group, while significant heterogeneity was not observed at RMS HOAs (*I*^2^ = 0%), RMS SA (*I*^2^ = 0%), and RMS coma (*I*^2^ = 16%) (Fig. 5).

### Heterogeneity analysis

The leave-one-out method indicated that after exclusion of the study by Jing, Yan & Wan (2025) and Paune et al. (2021) heterogeneity for both outcome measures (AE at 6 months and AE at 24 months) was reduced, with *I*^2^ values of 29% and 0%, respectively, suggesting that the studies by Jing, Yan & Wan (2025) and Paune et al. (2021) were the primary source of heterogeneity for these two outcomes. However, the leave-one-out method for the outcome measure of TZD indicated that its heterogeneity was not attributable to any single included study (Fig. S1).

Therefore, meta-regression was conducted to explore the source of heterogeneity. Previous studies have demonstrated that, apart from BOZD, other lens design parameters can influence TZD (Yang et al., 2021; Li et al., 2025a; Li et al., 2025b; Li et al., 2025c). Accordingly, the present research categorized the included studies according to three methodological aspects: study design (RCT and cohort study), lens design (KATT BE Free, CRT, DRL/Euclid, CRT/Euclid), and follow-up duration (1 month, 12 months, 24 months, or unreported). Meta-regression analysis revealed that lens design was the primary source of heterogeneity in the outcome measure of TZD (*p* = 0.015). Based on the results of the meta-regression analysis, this study stratified the subjects into four subgroups (KATT BE Free, CRT, DRL/Euclid, CRT/Euclid) according to lens design. Subgroup analysis revealed that, after grouping by lens design, no significant heterogeneity was observed within each subgroup (all *I*^2^ < 50%), and the findings also indicated differences in TZD among the various lens designs (Fig. S2, Table 3).

### Sensitivity analysis

The sensitivity analysis, conducted using the leave-one-out method, demonstrated that the study’s findings were robust and consistent (Fig. S3).

### Publication bias analysis

Funnel plots for AE during the 12-month and 24-month follow-up periods, as well as for RMS HOAs, RMS SA, and RMS coma, were symmetric, whereas those for AE at 6 months and TZD periods were asymmetric (Figs. S4–S5). The results of Egger’s test indicated that, except for AE at 6 months (*P* = 0.0485), the possibility of publication bias was low for the remaining outcome measures (each *P* > 0.05). Results from the non-parametric trim-and-fill method showed that, although publication bias might exist for AE at 6 months and TZD, its impact on the pooled results was relatively small (Fig. S6, Tables 4–5).

## Discussion

Ortho-K lenses, as a special type of corneal contact lenses, have been reported to delay AE in myopic children as early as 2005 (Cho, Cheung & Edwards, 2005). Even children with high myopia can achieve better myopia control through Ortho-K lens wear (Charm & Cho, 2013). Studies suggest that the efficacy of Ortho-K lenses in controlling childhood myopia may stem from the peripheral retinal myopic shift they induce (Kang & Swarbrick, 2011; Gonzalez-Meijome et al., 2016; Queirós, Pinheiro & Fernandes, 2025). AE is influenced more by the peripheral than the foveal retina (Flitcroft, 2012). Peripheral hyperopic defocus stimulates retinal neurons and promotes AE, whereas myopic defocus exerts the opposite effect (Russo et al., 2022; Dhiman et al., 2022). Imposing relative myopic defocus on the peripheral retina is thus an effective strategy to inhibit AE (Arumugam et al., 2014; Smith, Hung & Huang, 2009). The reverse geometry design of Ortho-K lenses remodels the cornea during wear (Vincent et al., 2021), producing annular mid-peripheral steepening and increasing peripheral myopic defocus (Vincent et al., 2021; Hiraoka, 2022). This has been proposed as a plausible mechanism underlying the ability of Ortho-K lenses to slow myopia progression (Kang & Swarbrick, 2011; Gonzalez-Meijome et al., 2016; Nti & Berntsen, 2020).

**Table 3:** Meta-regression results of TZD and RMS coma.

Outcome	Covariates	Coefficient	Std. err.	*z*	P >—*z*—	[95% conf. interval]
TZD	Study design	−0.184	0.176	−1.05	0.295	−0.529 to 0.161
	Follow-up duration	0.085	0.088	0.97	0.333	−0.087 to 0.258
	Lens design	−0.352	0.144	−2.44	0.015	−0.635 to −0.069
	_cons	0.032	0.241	0.13	0.894	−0.44 to 0.504

**Notes.**

TZD treatment zone diameter

**Table 4:** Results of Egger’s test for each outcome indicator.

Outcome	beta1	SE of beta1	*t*	*P* >—*t*—
AE at 6 m	−2.48	1.066	−2.33	0.0485
AE at 12 m	0.3	0.486	0.61	0.5523
AE at 24 m	−0.1	3.277	−0.03	0.9799
TZD	−2.36	1.087	−2.17	0.0729
RMS HOAs	0.22	0.685	0.32	0.7674
RMS SA	−1.46	1.345	−1.09	0.3903
RMS coma	1.02	1.995	0.51	0.6996

**Notes.**

AE axial elongation TZD treatment zone diameter RMS root mean square HOAs higher-order aberrations SA spherical aberration

**Table 5:** Nonparametric trim-and-fill analysis of publication bias.

Outcome	Studies	Mean diff.	[95% conf. interval]
AE at 6 m	Observed	−0.067	−0.088 to −0.046
	Observed + Imputed	−0.059	−0.081 to −0.037
TZD	Observed	−0.497	−0.644 to −0.350
	Observed + Imputed	−0.476	−0.621 to −0.331

**Notes.**

AE Axial elongation TZD treatment zone diameter

However, the peripheral retinal myopic shift induced by Ortho-K lenses does not fully account for their myopia-control mechanism. Studies report a positive correlation between post-wear ocular aberration changes and the degree of myopia correction (Hiraoka et al., 2007). As the central cornea flattens and refractive error decreases, HOAs, particularly SA and coma, increase markedly (Stillitano et al., 2007). Among optical parameters, coma change is the strongest predictor of AE inhibition (Hiraoka et al., 2015), with third-order aberrations and coma further confirmed as key factors related to AE (Kim et al., 2019). In children receiving Ortho-K, increased corneal HOAs (*e.g.*, horizontal coma, trefoil) are associated with slower myopia progression (Yoo et al., 2020). Ortho-K elevates Zernike coefficients, producing SA-dominant HOA alterations that may retard AE (Lau et al., 2020).

While these data link Ortho-K, induced optical changes to delayed myopia progression, some studies disagree. Santodomingo-Rubido et al. (2017) found significant corneal aberration changes after short-term and long-term wear, while no significant association with AE over 2 years. Conversely, Huang et al. (2022a) and Huang et al. (2022b) reported that although peripheral defocus soft contact lenses (PDSCLs) reduced myopia, their efficacy was inferior to Ortho-K, which induced greater HOAs changes despite less peripheral defocus, highlighting HOAs as the key to the Ortho-K group’s superior control. A further study (Liu et al., 2023) using spectacle lenses showed that adding cylindrical annular refractive elements to increase HOAs slowed AE, indirectly supporting the hypothesis that elevated HOAs may contribute to the protective effect of Ortho-K lenses.

In terms of myopia control efficacy, Cho, Cheung & Edwards (2005) in Hong Kong reported that conventional Ortho-K lenses reduced annual AE to 0.16 mm/y *versus* 0.34 mm/y with single-vision spectacles, corresponding to a control efficacy of approximately 52%. Pauné et al. (2015) in Europe observed an improvement in efficacy of about 38% relative to single-vision spectacles. Fang et al. (2022) in mainland China found that conventional Ortho-K lenses (0.34 mm/y) and PDSCLs (0.31 mm/y) demonstrated comparable efficacy, and both outperformed single-vision spectacles (0.45 mm/y). A meta-analysis indicated that conventional Ortho-K lenses were associated with a reduction of 0.19 mm in AE after one year and 0.27 mm after two years of wear. It decreased refractive error by −0.27D and −0.66D, respectively (Prousali et al., 2019), with efficacy equivalent to that of PDSCLs in slowing myopia progression (Fan et al., 2024).

Recently, multiple clinical studies (Li et al., 2023a; Li et al., 2023b; Li et al., 2023c; Li et al., 2023d; Xu et al., 2023; Paune et al., 2021; Guo et al., 2023a; Guo et al., 2023b) have shown that reducing the BOZD of Ortho-K lenses may potentially enhance the efficacy of myopia control in children and adolescents. The results of the present study indicate that, compared with conventional Ortho-K lenses, Ortho-K lenses with a smaller BOZD design reduce AE by 0.07 mm at 6 months and 0.12 mm at 12 months of follow-up.

Regarding this enhanced myopia control effect, some studies suggest that it may be related to alterations in TZD and HOAs. Li et al. (2023a), Li et al. (2023b), Li et al. (2023c) and Li et al. (2023d) compared Ortho-K lenses with different BOZD designs in children and found that TZD was the optimal predictor of AE, with AE limited to < 0.2 mm/year when TZD < 3.82 mm. This finding, that smaller TZD reduces AE, has been corroborated elsewhere Guo et al. (2023a), Guo et al. (2023b), Li et al. (2023a), Li et al. (2023b), Li et al. (2023c) and Li et al. (2023d). Li et al. (2023a), Li et al. (2023b), Li et al. (2023c) and Li et al. (2023d) compared 5.0 mm and 6.2 mm BOZD Ortho-K lenses in children and found that the smaller BOZD yielded a smaller TZD and a steeper relative corneal power distribution within the pupillary area, leading to a greater increase in total corneal HOAs and horizontal coma (Z31) and, consequently, a somewhat enhanced myopia control effect. Guo et al. (2023a) and Guo et al. (2023b) demonstrated that reducing the BOZD not only led to greater changes in HOAs and coma but also exhibited an increasing trend in the alteration of SA. Interestingly, Tan et al. (2025) challenged the notion that reducing BOZD increases HOAs, reporting no significant difference in HOA changes across BOZD designs. This disagreement has sparked debate over how smaller BOZD Ortho-K lenses enhance the efficacy of myopia control.

From the perspective of meta-analyses, Zhou et al. (2024) reported that Ortho-K lenses with a smaller BOZD slowed the progression of childhood myopia. Still, the effect was evident only in the first 6 months, with AE values of −0.11 mm at 6 months and −0.12 mm at 12 months. Additionally, limited to five included studies, the stability and publication bias were not verified.

Gu et al. (2024) also found that smaller BOZD reduced AE, while combining disparate follow-up durations and including only seven studies reduced statistical power. More recently, Yang et al. (2025) expanded the evidence base and preliminarily compared AE, TZD, and other parameters across follow-up periods, finding that smaller BOZD further reduced TZD and increased HOAs. However, the failure to elucidate the sources of heterogeneity necessitated the use of standardized mean differences (SMD), which reduced interpretability and precision. Additionally, the lack of further analysis of SA and coma changes within HOAs precluded an adequate resolution of prior controversies, limiting the study’s applicability.

This meta-analysis compared Ortho-K lenses with different BOZD on AE, TZD, and RMS HOAs, SA, and coma in children. We pioneered the use of weighted mean differences for 24-month AE and RMS HOAs, SA, and coma, enhancing clinical interpretability, precision, and applicability. Although some outcomes showed heterogeneity, the leave-one-out method revealed that the studies by Jing, Yan & Wan (2025) and Paune et al. (2021) accounted for the heterogeneity in AE at 6 months and AE at 24 months, respectively. Sensitivity analysis showed minor changes in the pooled effect size after exclusion of studies (AE at 6 months: −0.07 *vs* −0.07 mm; AE at 24 months: −0.14 *vs* −0.16 mm). However, the overall results were not materially altered, supporting the robustness of the outcome measures in these two meta-analyses. For TZD, no single study explained the heterogeneity, so meta-regression was used. With more included studies than prior meta-analyses, we identified non-BOZD lens design differences, including reverse curve, alignment curve, and peripheral curve, as a potential source. Recent studies (Li et al., 2025a; Li et al., 2025b; Li et al., 2025c) have reported that aspherical lenses produce smaller TZD than spherical lenses under the same BOZD, suggesting that other design optimizations besides modifying BOZD can enhance the efficacy of myopia control in children. Furthermore, the results of this meta-analysis provide a partial explanation for the previous controversy regarding whether reducing BOZD alters HOAs, SA, or coma. We found that smaller BOZD designs increased HOAs by 0.20 µm, SA by 0.17 µm, and coma by 0.10 µm in children, a response that diverges from that of Tan et al. (2025), Li et al. (2023a), Li et al. (2023b), Li et al. (2023c) and Li et al. (2023d) but aligns with Guo et al. (2023a) and Guo et al. (2023b).

It should be noted that, while reducing the BOZD currently offers improved myopia control efficacy, the concomitant decrease in TZD and increase in HOAs may compromise visual quality. Although (Li et al., 2023a; Li et al., 2023b; Li et al., 2023c; Li et al., 2023d) did not observe subjective visual acuity differences among groups with different BOZD, a subset of participants still reported a reduction in subjective vision. A short-term study further revealed that a smaller BOZD design was associated with decreased contrast sensitivity in wearers (Carracedo et al., 2019). In a long-term investigation, Tang et al. (2024) demonstrated that, despite comparable subjective visual acuity between groups, the smaller BOZD group exhibited reduced contrast sensitivity at low spatial frequencies, a change potentially attributable to alterations in TZD and HOAs. Therefore, although reducing BOZD may enhance the efficacy of myopia control, better balancing visual quality and myopia control efficacy remains a focus of future research, and it is also necessary and meaningful to pay attention to and explore how changes in visual quality among myopic children under the influence of BOZD affect their long-term learning and daily life.

It is noteworthy that current studies investigating the differential effects of Ortho-K lenses for childhood myopia control have been primarily confined to mainland China and Hong Kong, with scarce data from non-Asian populations. This likely reflects the persistently high prevalence of myopia in China (Zhu et al., 2023) and its sharp rise during the COVID-19 pandemic (Liu et al., 2025; Najafzadeh et al., 2023; Li et al., 2022), which intensified local demand to optimize Ortho-K efficacy. Consequently, post-2023 studies began evaluating smaller BOZD designs, which explains why those included here started in 2023. Before that, BOZD optimization attracted little attention, and relevant evidence was sparse. Notably, a similar pattern has emerged in the field of peripheral defocus-modifying spectacle lenses for pediatric myopia control (Li et al., 2025a; Li et al., 2025b; Li et al., 2025c; Han et al., 2026; Su et al., 2025). Nevertheless, the broader generalizability of the study should not be overlooked. Future studies are warranted to encompass diverse regions worldwide in order to minimize potential confounding factors arising from regional and ethnic differences, which also represents a limitation of the present investigation.

In addition to being regionally limited, this study has several other limitations: (1) Although AE at 24 months was included as an outcome measure in the meta-analysis, the number of eligible studies remains relatively small, and it is still necessary to conduct multicenter randomized controlled trials with longer follow-up durations (≥24 months) in the future. (2) Further investigation is needed into the potential effects of Ortho-K lenses with smaller BOZD on children’s long-term learning and daily life, to balance their myopia control benefits against changes in subjective and objective visual quality.

## Conclusions

Our meta-analysis revealed that Ortho-K lenses with smaller BOZD exhibited less axial elongation in children compared with conventional Ortho-K lenses. This effect may be related to the reduction of TZD and the increase of HOAs caused by the smaller BOZD design.

##  Supplemental Information

10.7717/peerj.20928/supp-1 Supplemental Information 1 PRISMA checklist

10.7717/peerj.20928/supp-2 Supplemental Information 2 Search strategies modified

10.7717/peerj.20928/supp-3 Supplemental Information 3 Supplemental_Figure The leave-one-out method revealed that the studies by Jingand Pauné were the primary source of heterogeneity for these two outcomes. Figure S2. Subgroup analysis of the treatment zone diameter. Figure S3. The sensitivity analysis results indicate that the findings of each meta-analysis are highly stable. Figure S4. Funnel plots showing AE at 6 months (A) , AE at 12 months (B) , AE at 24 months (C) , and TZD (D) , respectively. Figure S5. Funnel plots showing HOAs (A) , SA (B) , and Coma (C) respectively. Figure S6. The non-parametric trim and fill method showed that, although publication bias might exist for AE at 6 months and TZD, its impact on the pooled results was relatively small

10.7717/peerj.20928/supp-4 Supplemental Information 4 Systematic Review andor Meta-Analysis Rationale (1).The rationale for conducting the systematic review / meta-analysis. (2). The contribution that it makes to knowledge in light of previously published related reports, including other meta-analyses and systematic reviews.

10.7717/peerj.20928/supp-5 Supplemental Information 5 Raw data for this meta-analysis, including data from AE, TZD, RMS HOAs, RMS SA, and RMS coma

## Abbreviations

AE: axial elongation
Ortho-K: Orthokeratology
HOAs: higher-order aberrations
SA: spherical aberration
BOZD: back optic zone diameters
PRISMA: Preferred Reporting Items for Systematic Reviews and Meta-Analyses
PROSPERO: International Prospective Register of Systematic Reviews
RCTs: randomized controlled trials
TZD: treatment zone diameter
RMS: root mean square
NOS: Newcastle-Ottawa Scale
MD: mean difference
CI: confidence interval

## Additional Information and Declarations

### Data Availability

The following information was supplied regarding data availability: This is a systematic review/meta-analysis.
