# Supplementary material for: Optical changes and axial elongation in children wearing orthokeratology lenses of smaller back optic zone diameter: a systematic review and meta-analysis
Source: PeerJ. 2026 Mar 11;14:e20928. doi: 10.7717/peerj.20928 (PMC12988729; doi:10.7717/peerj.20928)

**Supplemental File 2. Search strategies modified and records**

**Appendix.**

**Search strategies modified in PubMed (a), the Cochrane Library (b), Embase (c), CNKI (d), and Web of Science (e).**

**a. Search strategy in PubMed**

| # | Search syntax | Citations found |
| --- | --- | --- |
| 1 | (orthokeratologic procedures[MeSH Terms]) OR (orthokeratology[All Fields]) OR (ortho-k[All Fields]) OR (OK lens[All Fields]) OR (DRL[All Fields]) OR (CRT[All Fields]) OR (Euclid[All Fields]) OR (αOrtho-K[All Fields]) OR (KATT BE Free[All Fields]) |  |
| 2 | (myopia[MeSH Terms]) OR (near sight[All Fields]) OR (refractive errors[All Fields]) OR (nearsightedness[All Fields]) OR (myopia control[All Fields]) |  |
| 3 | (diameter[All Fields]) OR (BOZD[All Fields]) OR (treatment zone[All Fields]) OR (optical zone[All Fields]) |  |
| 4 | #1 AND #2 AND #3 | 164 |

**b. Search strategy in the Cochrane Library**

| # | Search syntax | Citations found |
| --- | --- | --- |
| 1 | MeSH myopia |  |
| 2 | nearsight |  |
| 3 | refractive errors |  |
| 4 | nearsightedness |  |
| 5 | myopia control |  |
| 6 | #1 OR #2 OR #3 OR #4 OR #5 |  |
| 7 | MeSH orthokeratologic procedures |  |
| 8 | Orthokeratology |  |
| 9 | ortho-k |  |
| 10 | OK lens |  |
| 11 | orthokeratology lens |  |
| 12 | DRL |  |
| 13 | CRT |  |
| 14 | Euclid |  |
| 15 | αOrtho-K |  |
| 16 | KATT BE Free |  |
| 17 | #7 OR #8 OR #9 OR #10 OR #11 OR #12 OR #13 OR #14 OR #15 OR #16 |  |
| 18 | diameter |  |
| 19 | BOZD |  |
| 20 | treatment zone |  |
| 21 | optical zone |  |
| 22 | #18 OR #19 OR #20 OR #21 |  |
| 23 | #6 AND #17 AND #22 | 50 |

**c. Search strategy in Embase**

| # | Search syntax | Citations found |
| --- | --- | --- |
| 1 | MeSH myopia |  |
| 2 | nearsight |  |
| 3 | refractive errors |  |
| 4 | nearsightedness |  |
| 5 | myopia control |  |
| 6 | #1 OR #2 OR #3 OR #4 OR #5 |  |
| 7 | MeSH orthokeratologic procedures |  |
| 8 | Orthokeratology |  |
| 9 | ortho-k |  |
| 10 | OK lens |  |
| 11 | orthokeratology lens |  |
| 12 | DRL |  |
| 13 | CRT |  |
| 14 | Euclid |  |
| 15 | αOrtho-K |  |
| 16 | KATT BE Free |  |
| 17 | #7 OR #8 OR #9 OR #10 OR #11 OR #12 OR #13 OR #14 OR #15 OR #16 |  |
| 18 | diameter |  |
| 19 | BOZD |  |
| 20 | treatment zone |  |
| 21 | optical zone |  |
| 22 | #18 OR #19 OR #20 OR #21 |  |
| 23 | #6 AND #17 AND #22 | 233 |

**d. Search strategy in CNKI**

| # | Search syntax | Citations found |
| --- | --- | --- |
| 1 | myopia |  |
| 2 | near sight |  |
| 3 | refractive errors |  |
| 4 | nearsightedness |  |
| 5 | #1 OR #2 OR #3 OR #4 |  |
| 6 | orthokeratologic procedures |  |
| 7 | Orthokeratology |  |
| 8 | ortho-k |  |
| 9 | OK lens |  |
| 10 | orthokeratology lens |  |
| 11 | #6 OR #7 OR #8 OR #9 OR #10 |  |
| 12 | diameter |  |
| 13 | BOZD |  |
| 14 | #12 OR #13 |  |
| 15 | #5 AND #11 AND #12 | 73 |

**e. Search strategy in Web of Science**

| # | Search syntax | Citations found |
| --- | --- | --- |
| 1 | myopia |  |
| 2 | nearsightedness |  |
| 3 | #1 OR #2 |  |
| 4 | Orthokeratology |  |
| 5 | ortho-k |  |
| 6 | #4 OR #5 |  |
| 7 | diameter |  |
| 8 | BOZD |  |
| 9 | #7 OR #8 |  |
| 10 | #3 AND #6 AND #9 | 163 |

| # | Search syntax | Citations found |
| --- | --- | --- |
| 1 | ((((ALL=(nearsight)) OR ALL=(myopia)) OR ALL=('refractive errors')) OR ALL=(nearsightedness)) AND ALL=(myopia control) |  |
| 2 | (((((((((ALL=('orthokeratologic procedures')) OR ALL=(Orthokeratology)) OR ALL=('ortho-k')) OR ALL=('OK lens')) OR ALL=(orthokeratology lens)) OR ALL=(DRL)) OR ALL=(CRT)) OR ALL=(Euclid)) OR ALL=(αOrtho-K)) OR ALL=(KATT BE Free) |  |
| 3 | (((ALL=(diameter)) OR ALL=(BOZD)) OR ALL=(treatment zone)) OR ALL=(optical zone) |  |
| 4 | #1 OR #2 OR #3 OR #4 | 116 |

Search Records

1.Embase search records


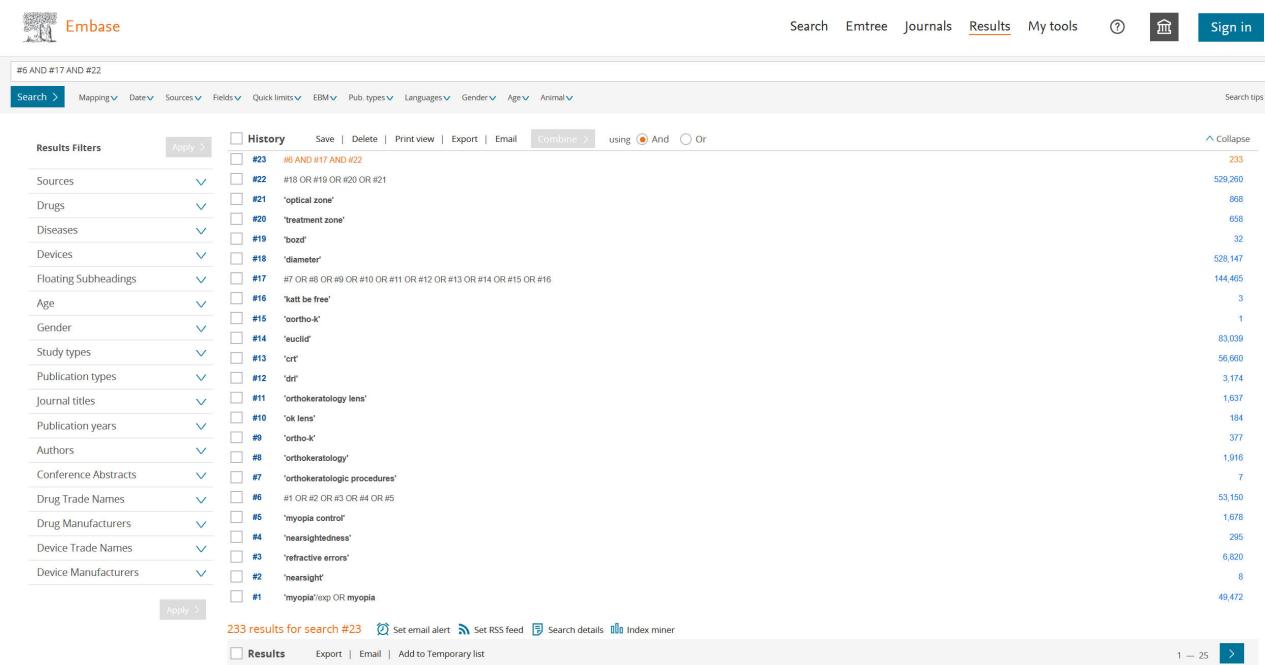


2.Pubmed search records


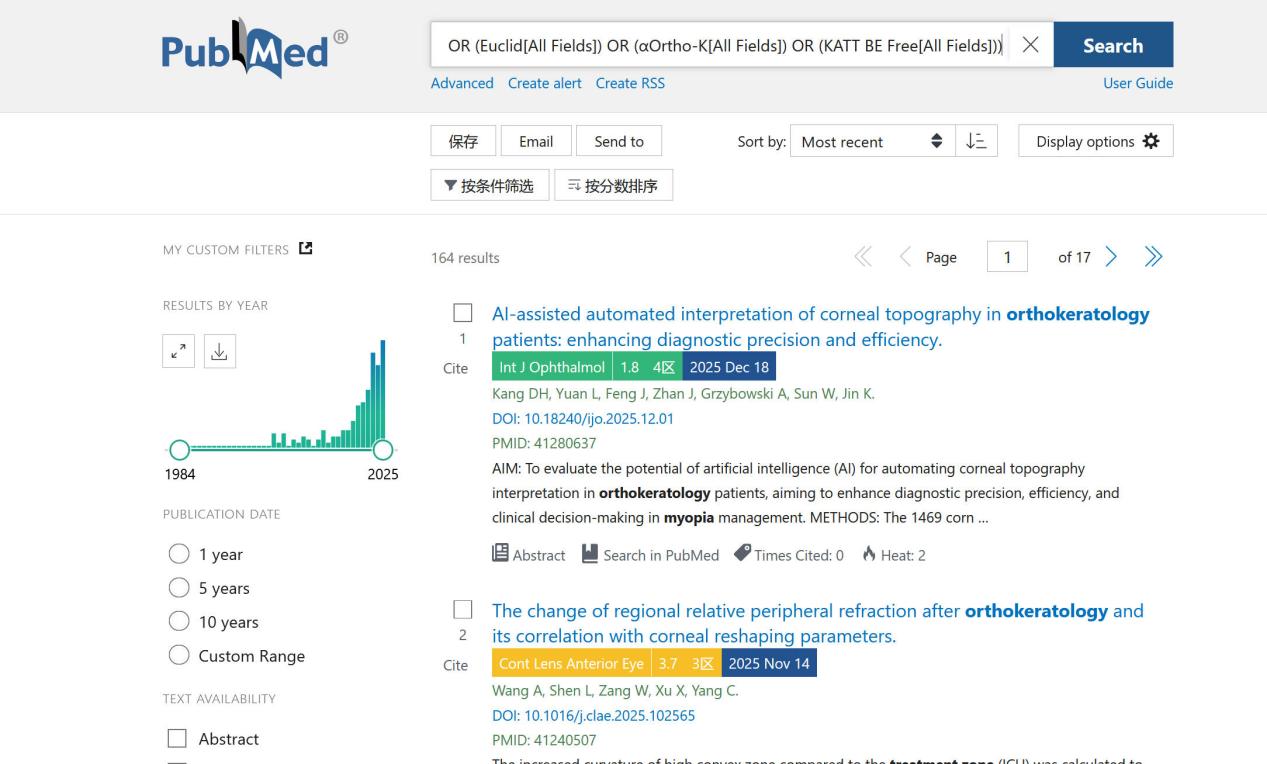


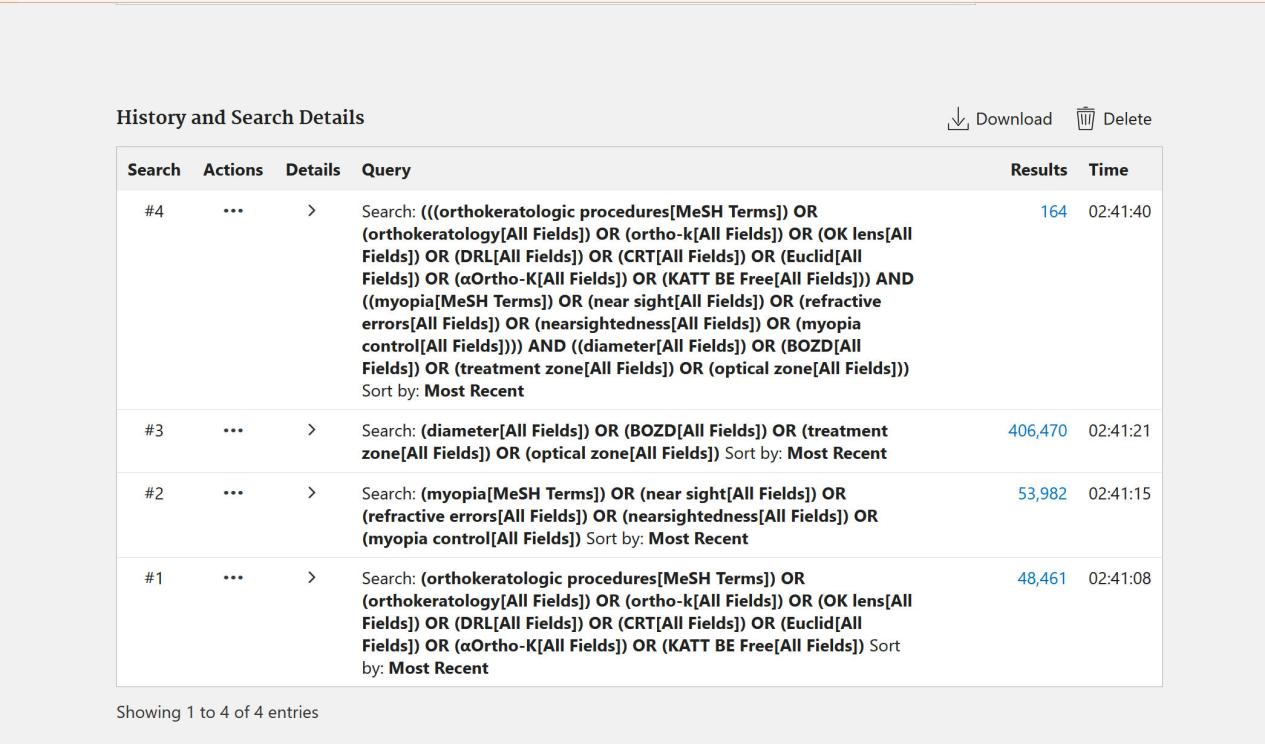


3. Web of science search records


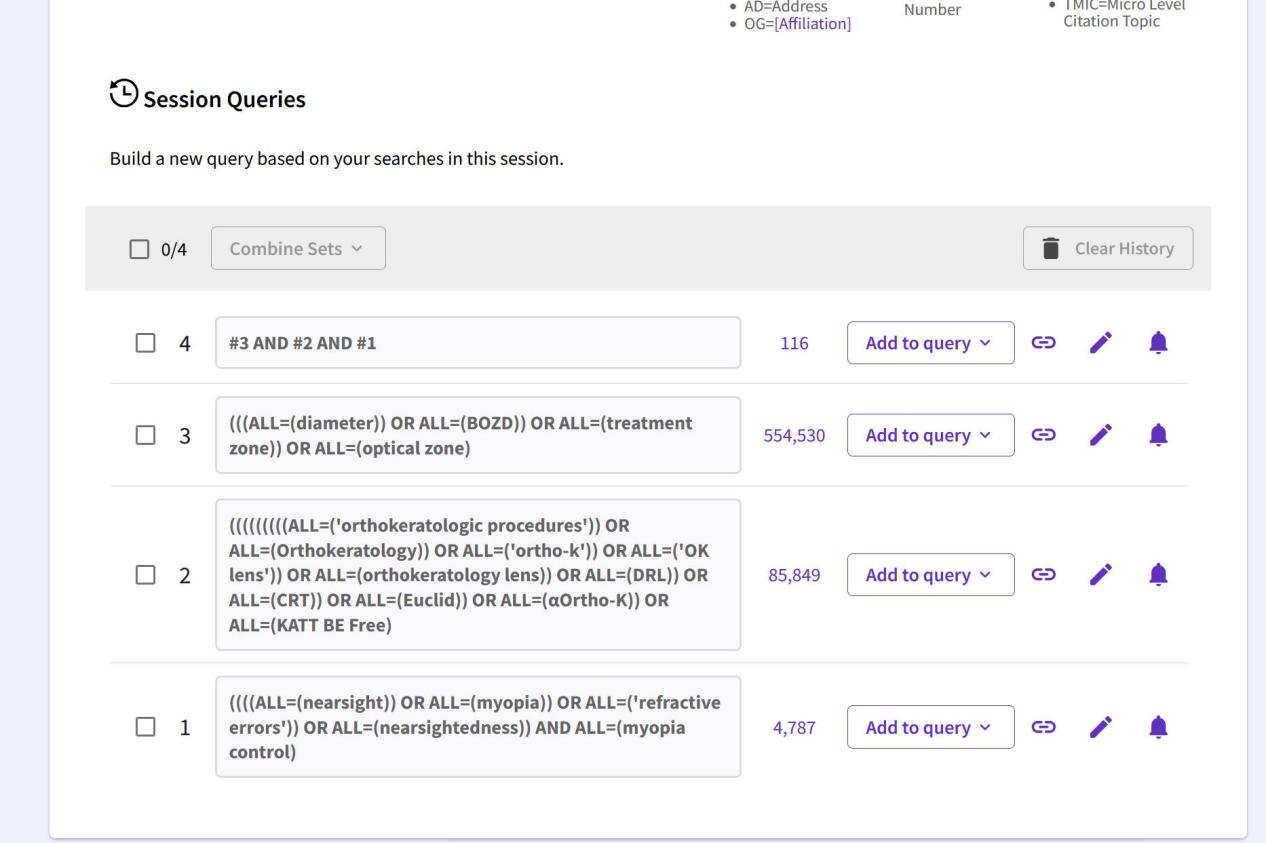


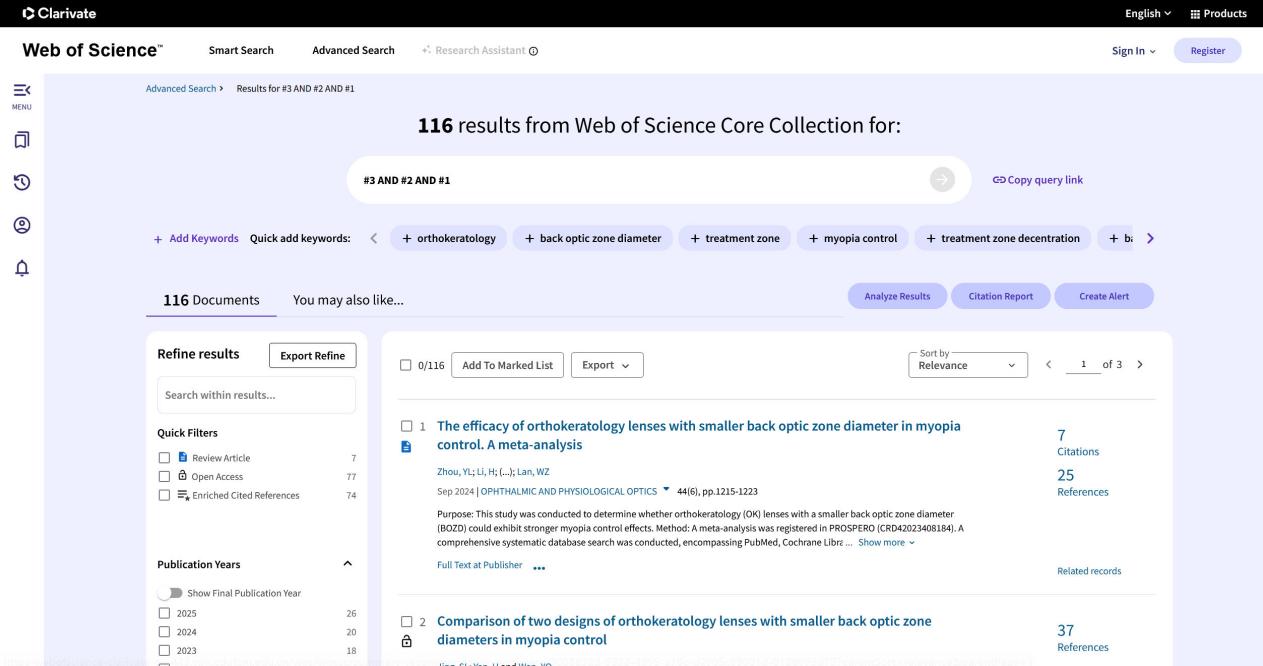


4.CNKI search records


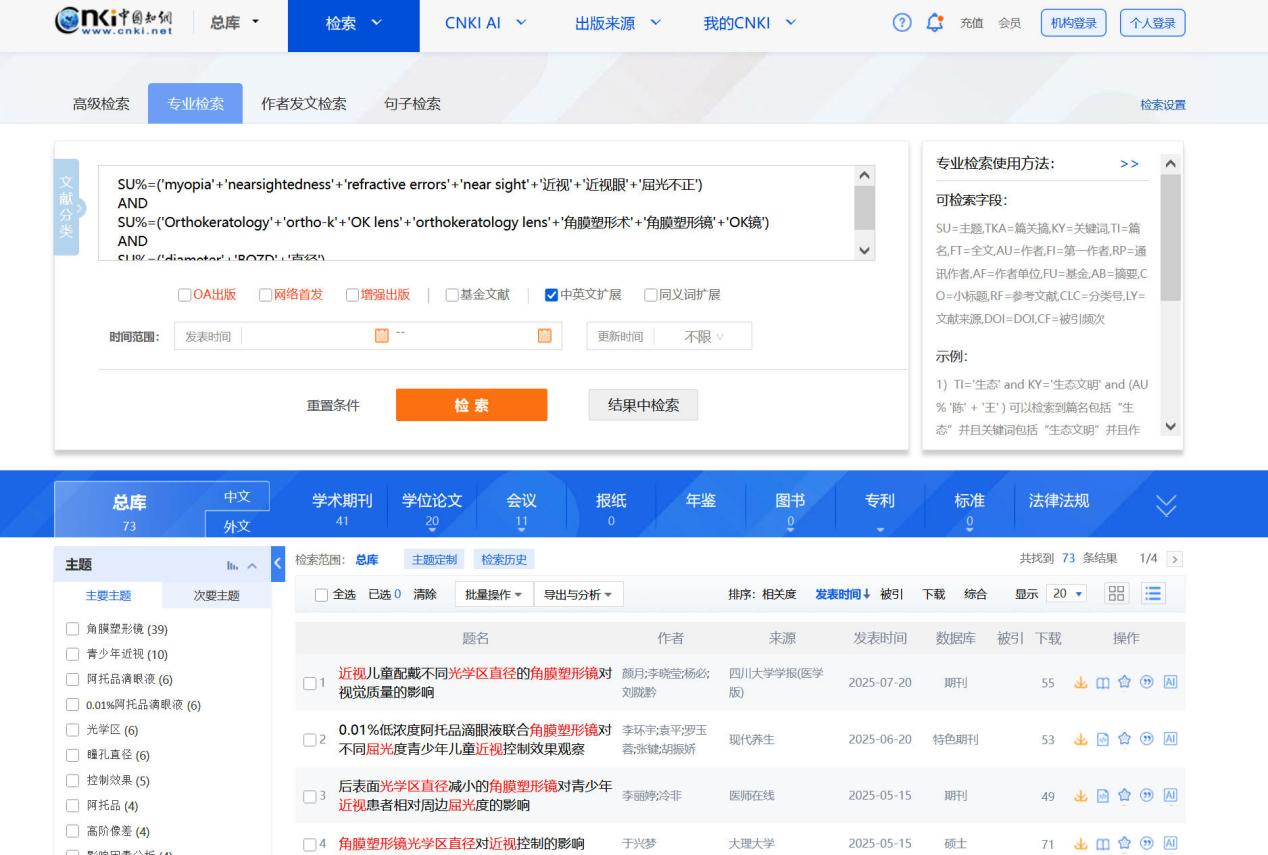


5.Cochrane Library search records


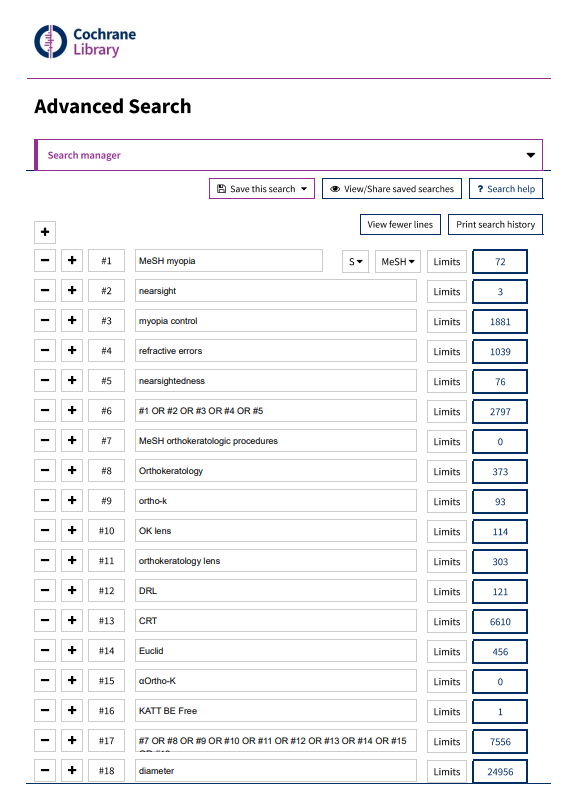


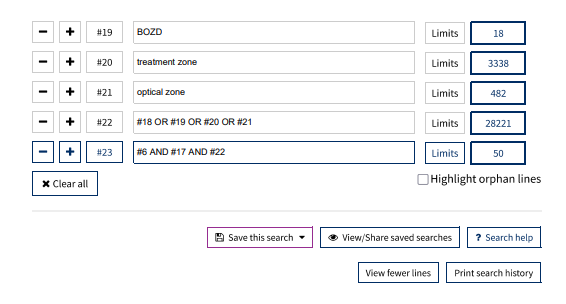

Supplement: Supplemental Information 2 [file peerj-14-20928-s002.docx]
