# Supplementary figures and images for: Optical changes and axial elongation in children wearing orthokeratology lenses of smaller back optic zone diameter: a systematic review and meta-analysis
Source: PeerJ. 2026 Mar 11;14:e20928. doi: 10.7717/peerj.20928 (PMC12988729; doi:10.7717/peerj.20928)

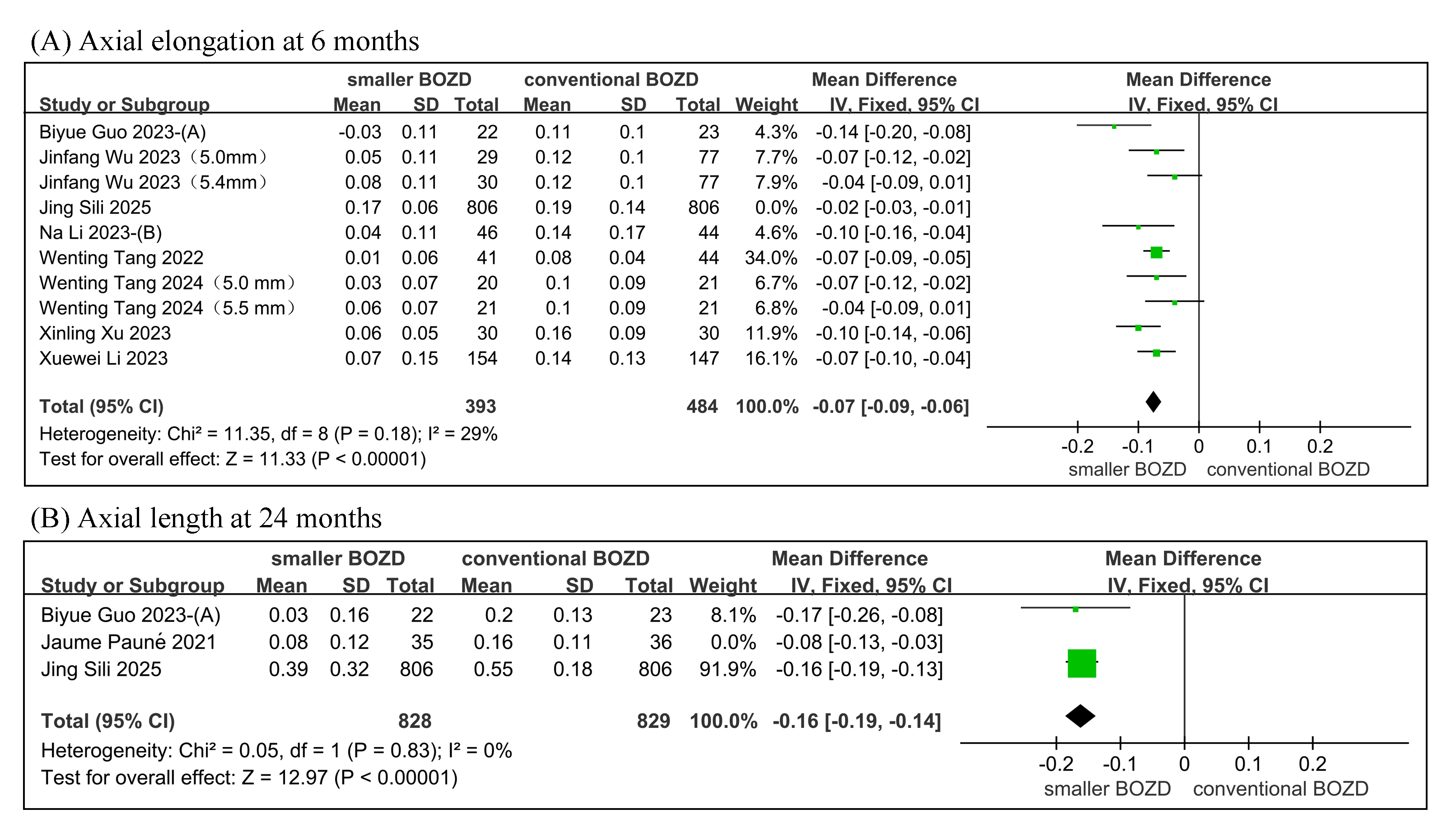

Supplement: Supplemental Information 3 — The leave-one-out method revealed that the studies by Jingand Pauné were the primary source of heterogeneity for these two outcomes. Figure S2. Subgroup analysis of the treatment zone diameter. Figure S3. The sensitivity analysis results indicate that the findings of each meta-analysis are highly stable. Figure S4. Funnel plots showing AE at 6 months (A) , AE at 12 months (B) , AE at 24 months (C) , and TZD (D) , respectively. Figure S5. Funnel plots showing HOAs (A) , SA (B) , and Coma (C) respectively. Figure S6. The non-parametric trim and fill method showed that, although publication bias might exist for AE at 6 months and TZD, its impact on the pooled results was relatively small [file peerj-14-20928-s003.zip › Figure S1 AE leave-one-out method.png]

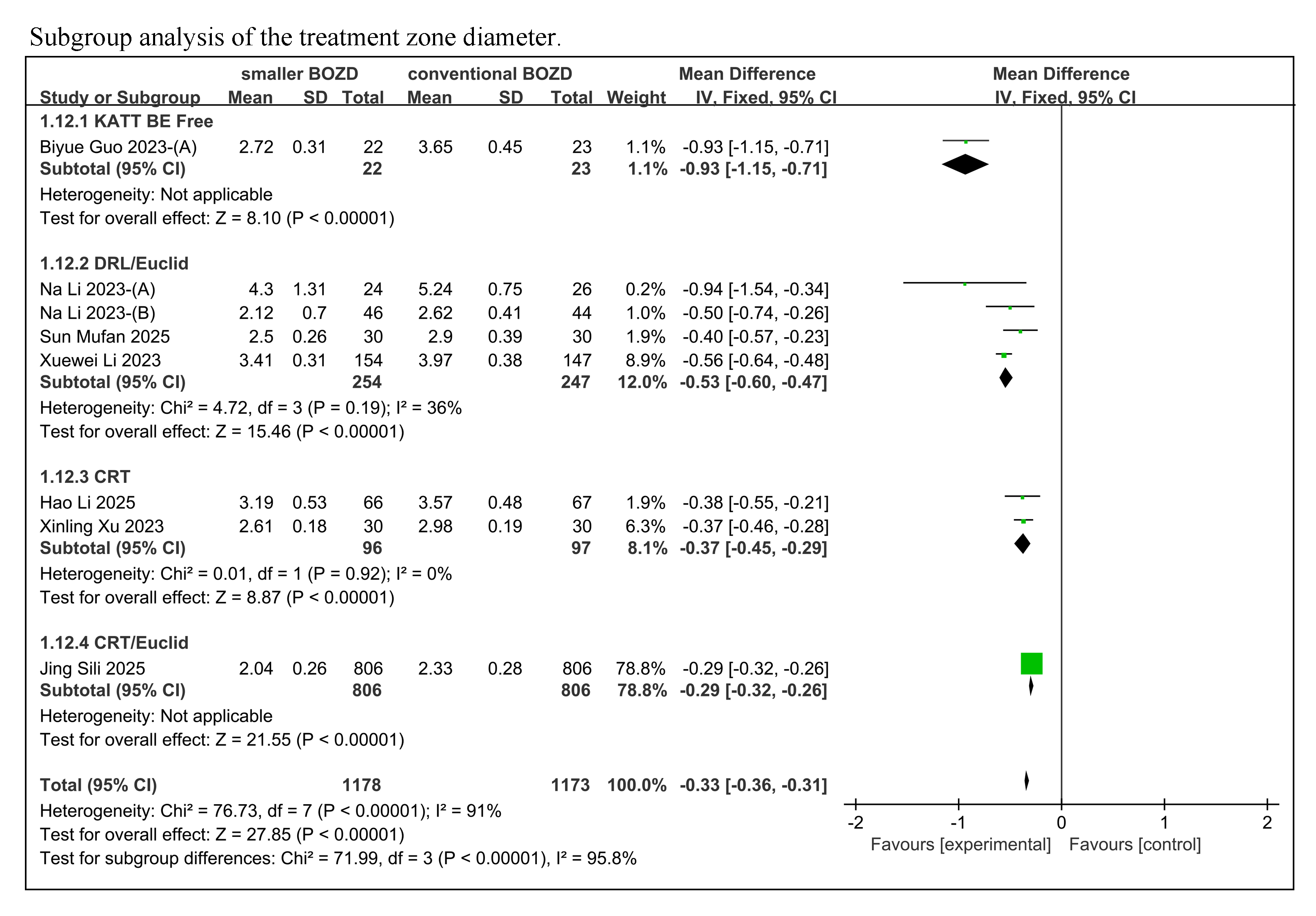

Supplement: Supplemental Information 3 — The leave-one-out method revealed that the studies by Jingand Pauné were the primary source of heterogeneity for these two outcomes. Figure S2. Subgroup analysis of the treatment zone diameter. Figure S3. The sensitivity analysis results indicate that the findings of each meta-analysis are highly stable. Figure S4. Funnel plots showing AE at 6 months (A) , AE at 12 months (B) , AE at 24 months (C) , and TZD (D) , respectively. Figure S5. Funnel plots showing HOAs (A) , SA (B) , and Coma (C) respectively. Figure S6. The non-parametric trim and fill method showed that, although publication bias might exist for AE at 6 months and TZD, its impact on the pooled results was relatively small [file peerj-14-20928-s003.zip › Figure S2 subgroup analysis.png]

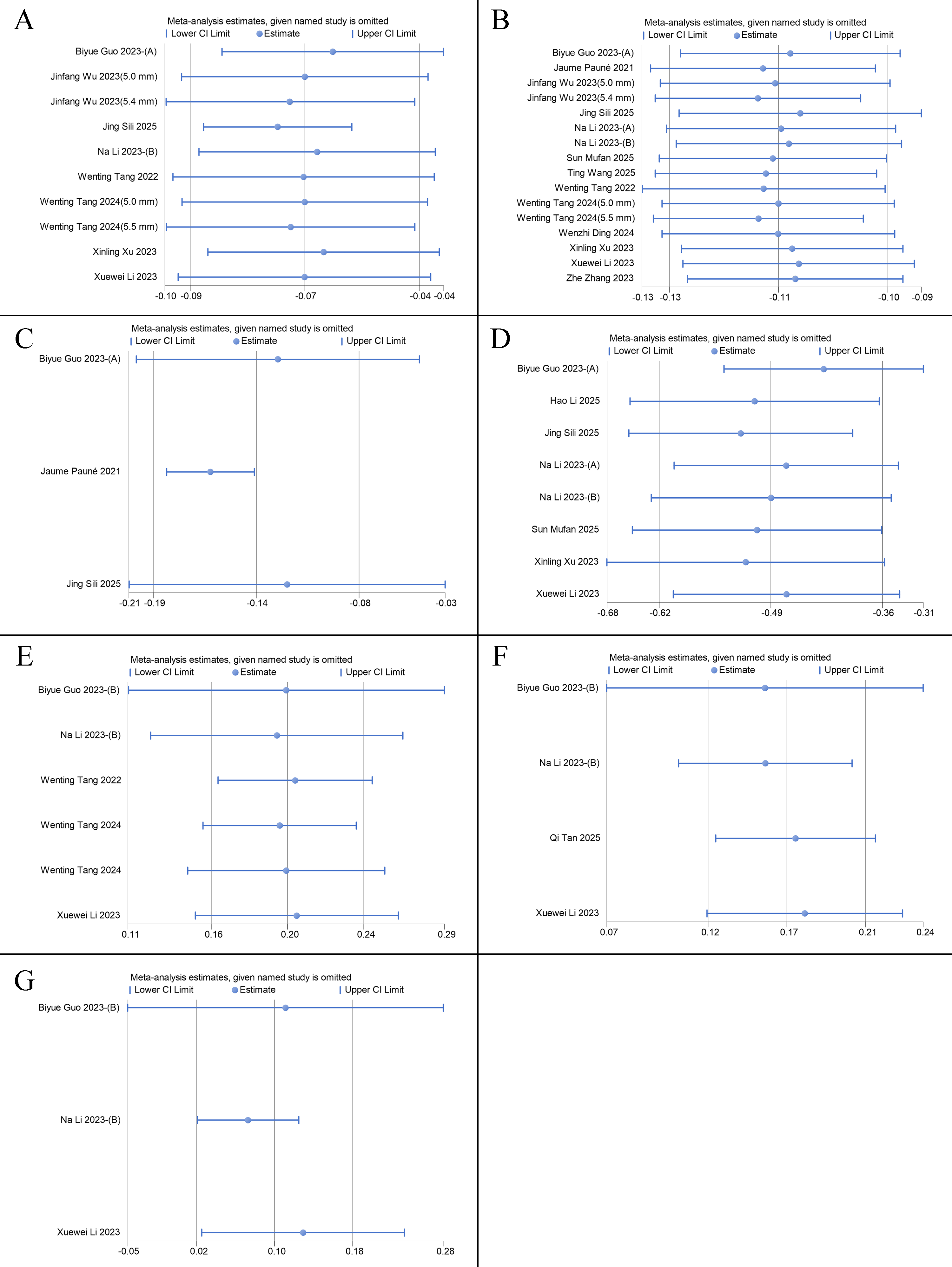

Supplement: Supplemental Information 3 — The leave-one-out method revealed that the studies by Jingand Pauné were the primary source of heterogeneity for these two outcomes. Figure S2. Subgroup analysis of the treatment zone diameter. Figure S3. The sensitivity analysis results indicate that the findings of each meta-analysis are highly stable. Figure S4. Funnel plots showing AE at 6 months (A) , AE at 12 months (B) , AE at 24 months (C) , and TZD (D) , respectively. Figure S5. Funnel plots showing HOAs (A) , SA (B) , and Coma (C) respectively. Figure S6. The non-parametric trim and fill method showed that, although publication bias might exist for AE at 6 months and TZD, its impact on the pooled results was relatively small [file peerj-14-20928-s003.zip › Figure S3. Sensitivity analysis.png]

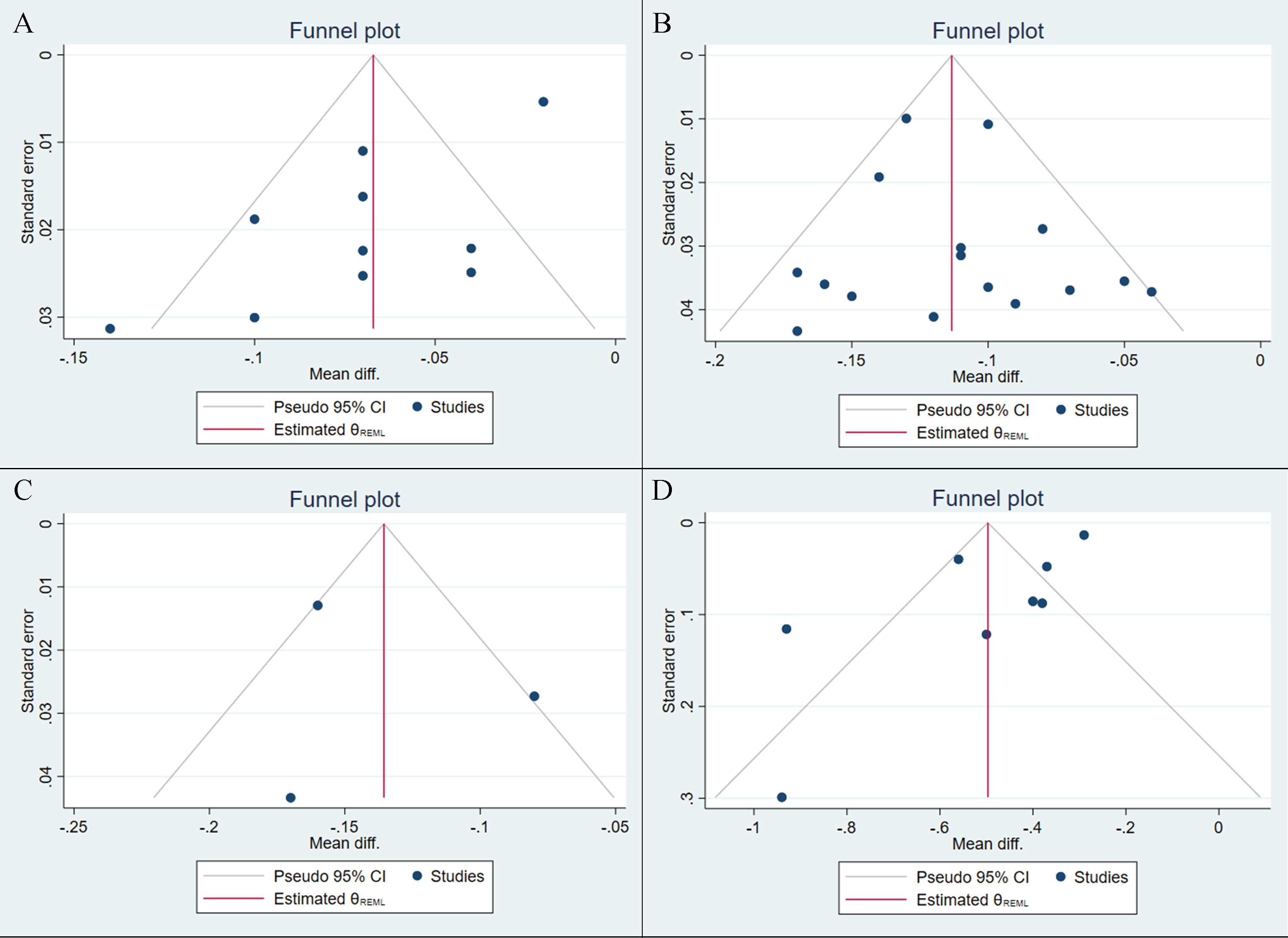

Supplement: Supplemental Information 3 — The leave-one-out method revealed that the studies by Jingand Pauné were the primary source of heterogeneity for these two outcomes. Figure S2. Subgroup analysis of the treatment zone diameter. Figure S3. The sensitivity analysis results indicate that the findings of each meta-analysis are highly stable. Figure S4. Funnel plots showing AE at 6 months (A) , AE at 12 months (B) , AE at 24 months (C) , and TZD (D) , respectively. Figure S5. Funnel plots showing HOAs (A) , SA (B) , and Coma (C) respectively. Figure S6. The non-parametric trim and fill method showed that, although publication bias might exist for AE at 6 months and TZD, its impact on the pooled results was relatively small [file peerj-14-20928-s003.zip › Figure S4.png]

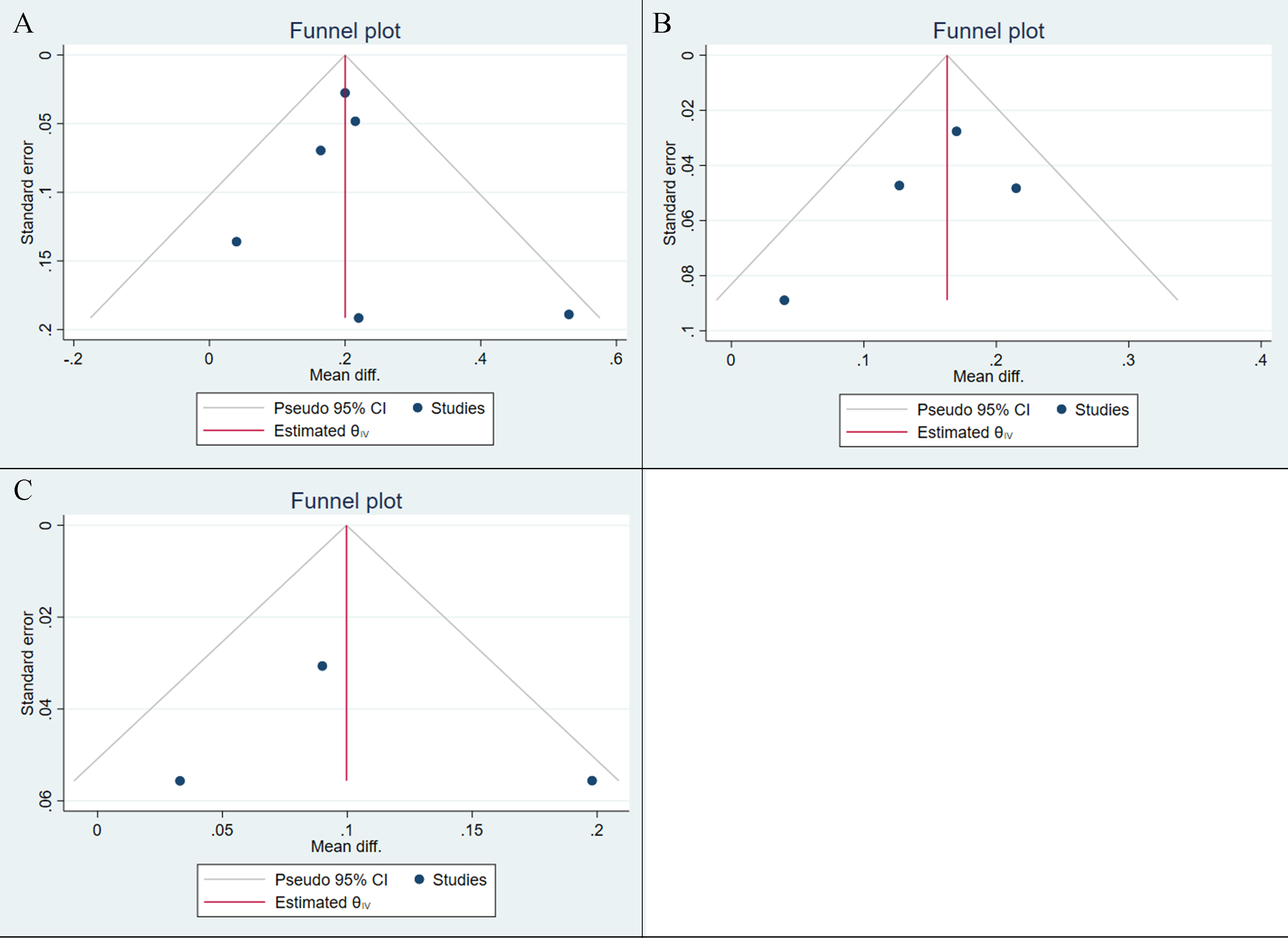

Supplement: Supplemental Information 3 — The leave-one-out method revealed that the studies by Jingand Pauné were the primary source of heterogeneity for these two outcomes. Figure S2. Subgroup analysis of the treatment zone diameter. Figure S3. The sensitivity analysis results indicate that the findings of each meta-analysis are highly stable. Figure S4. Funnel plots showing AE at 6 months (A) , AE at 12 months (B) , AE at 24 months (C) , and TZD (D) , respectively. Figure S5. Funnel plots showing HOAs (A) , SA (B) , and Coma (C) respectively. Figure S6. The non-parametric trim and fill method showed that, although publication bias might exist for AE at 6 months and TZD, its impact on the pooled results was relatively small [file peerj-14-20928-s003.zip › Figure S5.png]

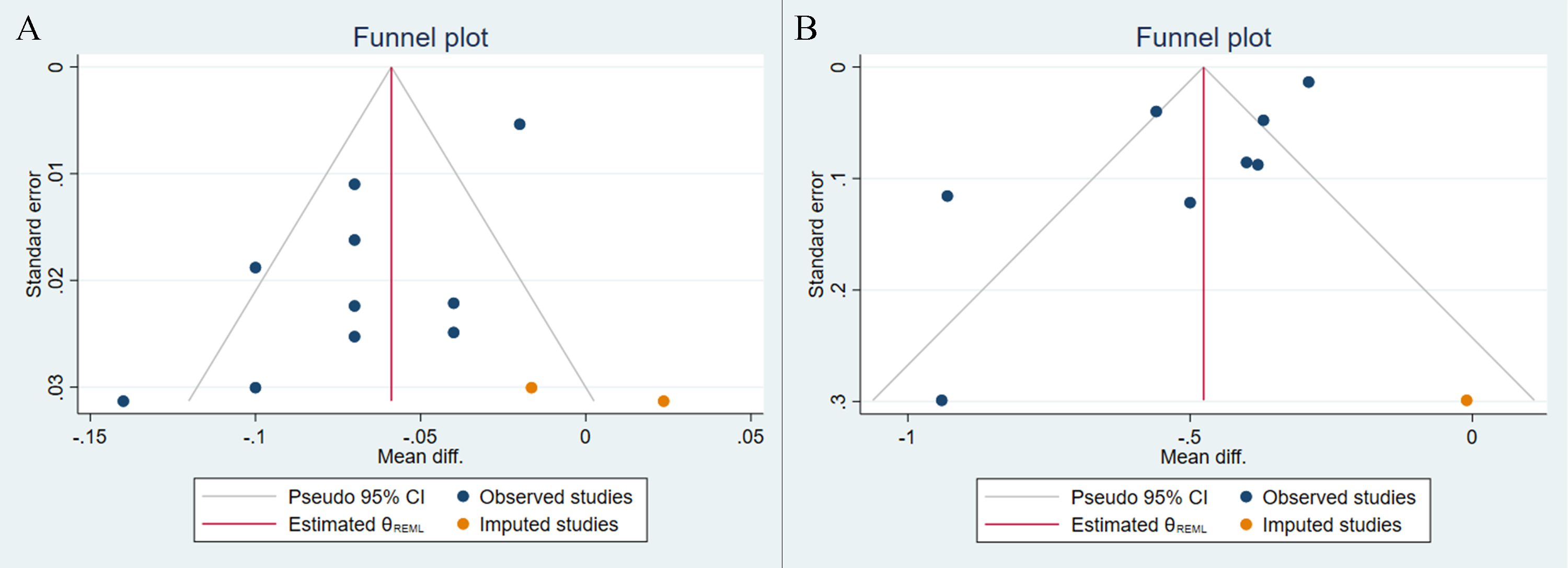

Supplement: Supplemental Information 3 — The leave-one-out method revealed that the studies by Jingand Pauné were the primary source of heterogeneity for these two outcomes. Figure S2. Subgroup analysis of the treatment zone diameter. Figure S3. The sensitivity analysis results indicate that the findings of each meta-analysis are highly stable. Figure S4. Funnel plots showing AE at 6 months (A) , AE at 12 months (B) , AE at 24 months (C) , and TZD (D) , respectively. Figure S5. Funnel plots showing HOAs (A) , SA (B) , and Coma (C) respectively. Figure S6. The non-parametric trim and fill method showed that, although publication bias might exist for AE at 6 months and TZD, its impact on the pooled results was relatively small [file peerj-14-20928-s003.zip › Figure S6.png]
