# Supplementary material for: Optical changes and axial elongation in children wearing orthokeratology lenses of smaller back optic zone diameter: a systematic review and meta-analysis
Source: PeerJ. 2026 Mar 11;14:e20928. doi: 10.7717/peerj.20928 (PMC12988729; doi:10.7717/peerj.20928)
Supplement: Supplemental Information 4 — (1).The rationale for conducting the systematic review / meta-analysis. (2). The contribution that it makes to knowledge in light of previously published related reports, including other meta-analyses and systematic reviews. [file peerj-14-20928-s004.docx]

**Systematic Review and/or Meta-Analysis Rationale**

**1.The rationale for conducting the systematic review / meta-analysis.**

In the clinical management of pediatric myopia, orthokeratology (Ortho-K) is widely recognized as an important intervention capable of effectively slowing myopic progression. Through a review of relevant literature, we became interested in the role of orthokeratology lenses with a small back optic zone diameter (BOZD) in inhibiting axial elongation in myopic children. Although existing studies provide evidence supporting the efficacy of small BOZD orthokeratology lenses in controlling axial elongation in children, we believe that current direct comparisons of orthokeratology lenses with varying BOZDs in delaying pediatric myopia remain insufficient. Moreover, unresolved controversies exist regarding the optical changes induced by small BOZD lenses across different studies, highlighting the need for updated evidence and reassessment of the role of a broader range of small BOZD orthokeratology lenses in pediatric myopia intervention.

**2. The contribution that it makes to knowledge in light of previously published related reports, including other meta-analyses and systematic reviews.**

Previously published meta-analyses and systematic reviews, such as those by Gu and Zhou et al., examined the effects of small BOZD Ortho-K lenses on axial length and refractive outcomes in myopic children. However, these studies were limited by a small number of included studies and insufficient sample sizes. Yang et al. expanded the scope of research by further comparing small BOZD Ortho-K lenses with conventional Ortho-K lenses in terms of Treatment Zone Diameter (TZD) and Higher-Order Aberrations (HOAs). Nevertheless, their meta-analysis did not analyze specific changes in spherical aberration and coma within HOAs, which remain contentious issues in the field. Additionally, their findings exhibited unexplained sources of heterogeneity and lacked necessary sensitivity analyses and assessments for publication bias.

Based on our review of the literature, we hypothesized that the myopia control efficacy of small BOZD Ortho-K lenses might be associated with TZD and specific HOAs parameters, particularly spherical aberration and coma. By expanding the number of included studies and total sample size, our study provided a more comprehensive comparison of key HOAs metrics, including spherical aberration and coma. Rigorous methodological approaches, such as sensitivity analysis, meta-regression, subgroup analysis, Egger`s test, and funnel plots, were employed to ensure the reliability and robustness of our conclusions.
